# Supplementary material for: Ag9GaSe6: high-pressure-induced Ag migration causes thermoelectric performance irreproducibility and elimination of such instability
Source: Nat Commun. 2022 May 27;13:2966. doi: 10.1038/s41467-022-30716-7 (PMC9142491; doi:10.1038/s41467-022-30716-7)
Supplement: Supplementary file 1 — Supporting Information [file 41467_2022_30716_MOESM1_ESM.pdf]

Supplementary Information for

Ag_9GaSe_6: High-pressure-induced Ag migration causes thermoelectric performance irreproducibility and elimination of such instability

Jing-Yuan Liu,[1] Ling Chen[1,2,*], and Li-Ming Wu[1,2,*]

[1]*Beijing Key Laboratory of Energy Conversion and Storage Materials, College of Chemistry, Beijing Normal University, Beijing 100875, People's Republic of China*

[2]*Center for Advanced Materials Research, Advanced Institute of Natural Sciences, Beijing Normal University, Zhuhai 519087, People's Republic of China*

* E-mail: chenl@bnu.edu.cn, wlm@bnu.edu.cn

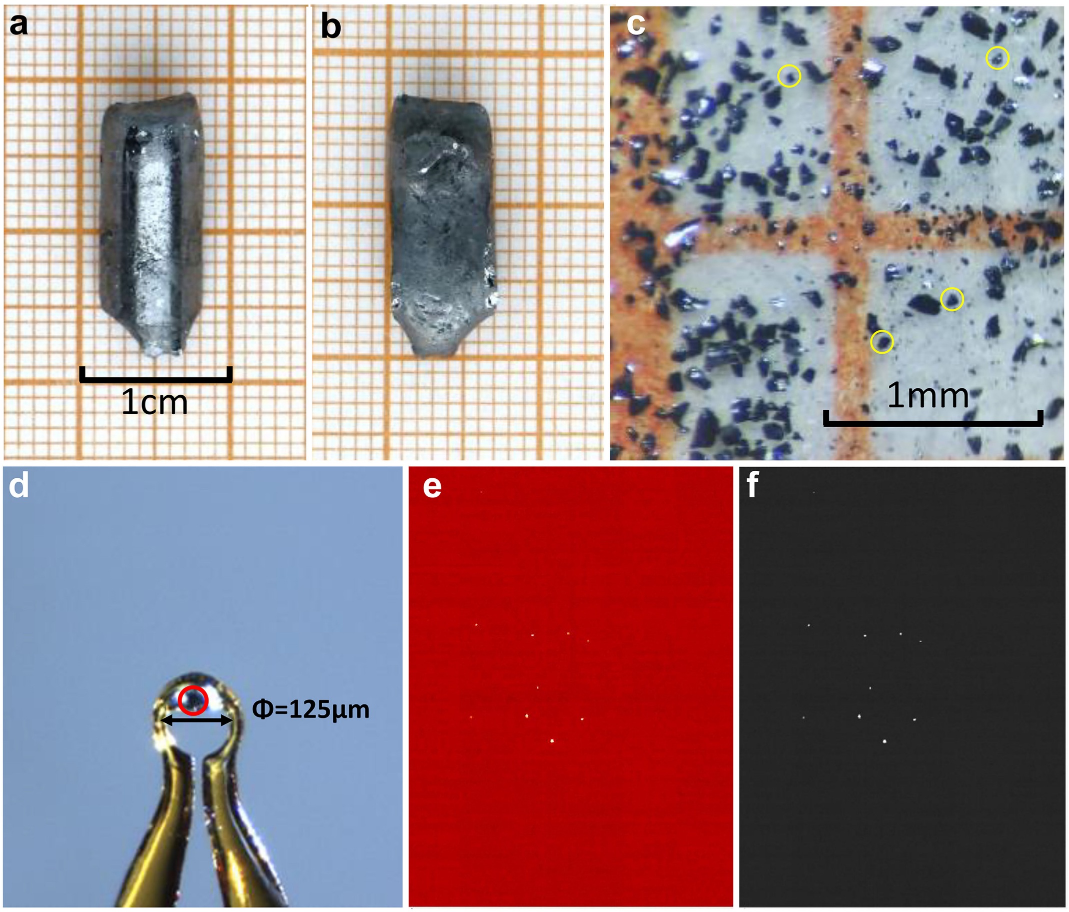

Supplementary Fig. 1. Single crystal picking and XRD data collecting process. **a**, **b**, The as-synthesized Ag_9GaSe_6 ingot product. **c**, The as-synthesized ingot was crushed and small single crystals (several are marked in yellow) are hand-picked to collect the single crystal diffraction data. **d**, The crystal with a size of tens of microns (marked in red) is set on the loop. **e**, A screenshot image of the clear and orderly diffractions during the single crystal diffraction data collection. **f**, In order to display the diffractions more clearly, we display the original diffraction pattern in grayscale.

Comparison between the measured and Dulong-Petit estimated C_p

As shown in Supplementary Fig. 2, the measured C_p (with an average of 0.29 J/g·K) is 10% higher than the Dulong-Petit estimated 0.2635 J/g·K, consisting well with the measured C_p reported[1]. The peak appearing around 330 K indicating a phase transitionin[2] in $Ag_{8.3}Cu_{0.7}GaSe_6$ sample was also observed. Remarkably, good consistency was observed on samples before- and after-the annealing treatment. (Supplementary Fig. 2)

The thermal conductivity (κ) and ZT of Ag_9GaSe_6 calculated by the measured C_p are compared with those calculated by Dulong-Petit C_p and ref. 1. As shown in Fig. 2c and g, due to the higher measured C_p than the Dulong-Petit estimated value, the corresponding κ is also overall higher than the later, with an average difference of ~ 10%. Accordingly, the overall ZT using experimental C_p (in Fig. 2d and h) is also averagely ~ 10% lower than the Dulong-Petit one.

Besides, although both $Ag_9GaSe_{5.5}Te_{0.5}$[3] and $Ag_{8.3}Cu_{0.7}GaSe_6$[2] use Dulong-Petit C_p, still we show the measured C_p-calculated κ and ZT in Supplementary Fig. 3 and 4. Obviously, except for few points with a difference of ~ 10%, the overall κ and ZT calculated by measured C_p are highly consistent with those calculated by Dulong-Petit C_p, indicating the accuracy and reasonableness of the Dulong-Petit estimation and the stability and reliability of our C_p measurement results.

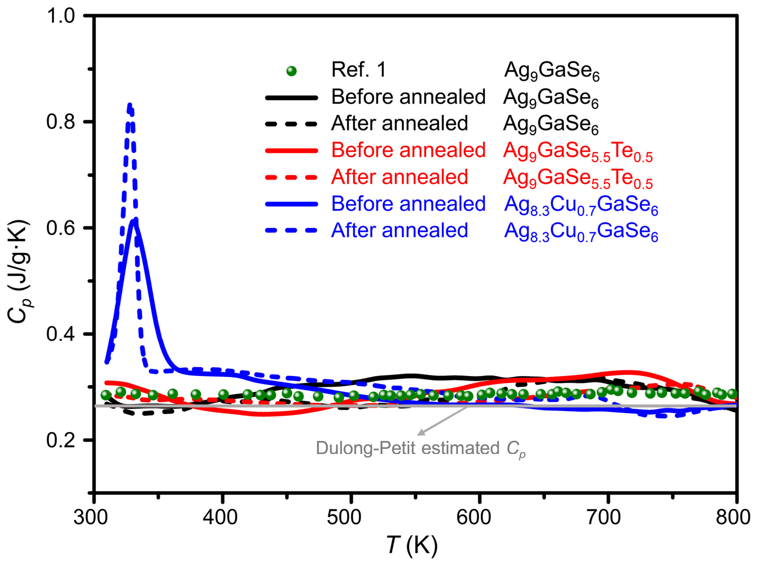

Supplementary Fig. 2. C_p-temperature curves of measured on the samples of Ag_9GaSe_6, $Ag_9GaSe_{5.5}Te_{0.5}$ and $Ag_{8.3}Cu_{0.7}GaSe_6$ before and after the annealing treatment. The measured C_p reported in ref. 1 and the C_p estimated by Dulong-Petit Law (~ 0.2635 J/g·K) are also showed.

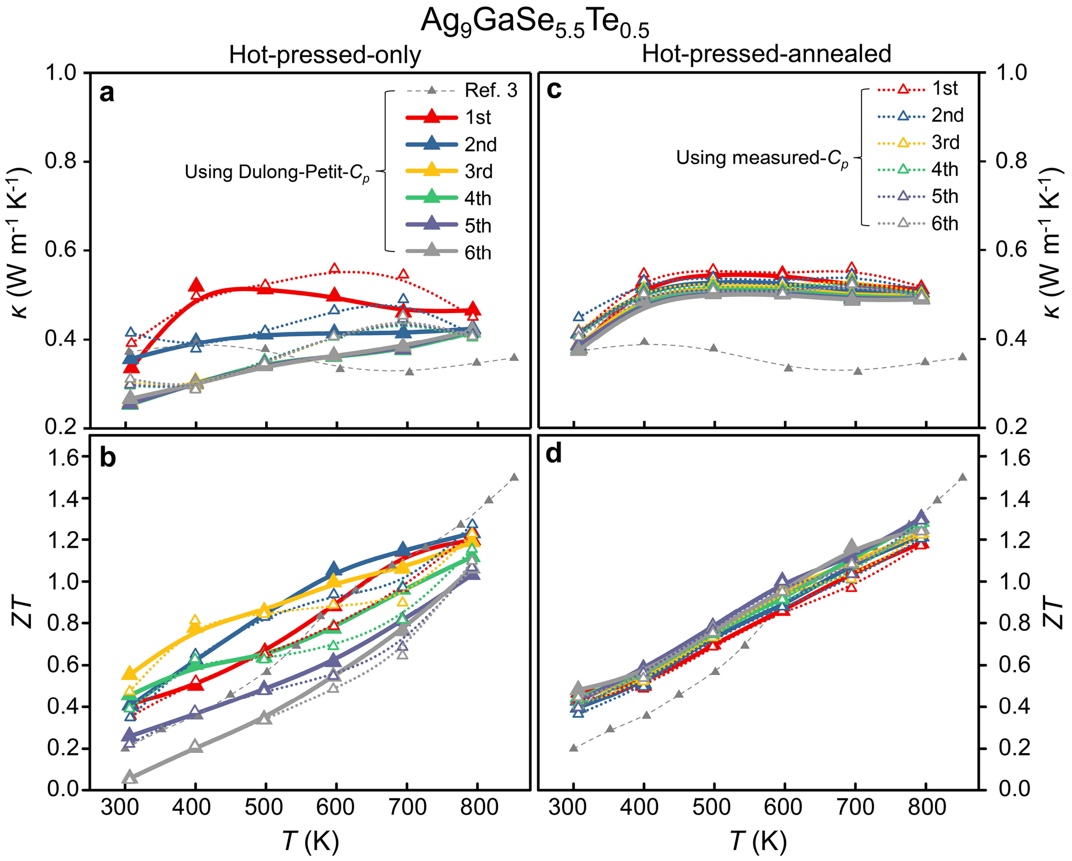

Supplementary Fig. 3. Temperature-dependent thermal conductivity (κ) and figure of merit (*ZT*) of $Ag_9GaSe_{5.5}Te_{0.5}$: **a, b,** hot-pressed-only and **c, d,** hot-pressed-annealed samples. The κ and *ZT* calculated by both the measured-C_p and Dulong-Petit-C_p are presented. Those reported in ref. 3 that are calculated by the Dulong-Petit-C_p are also presented for comparison.

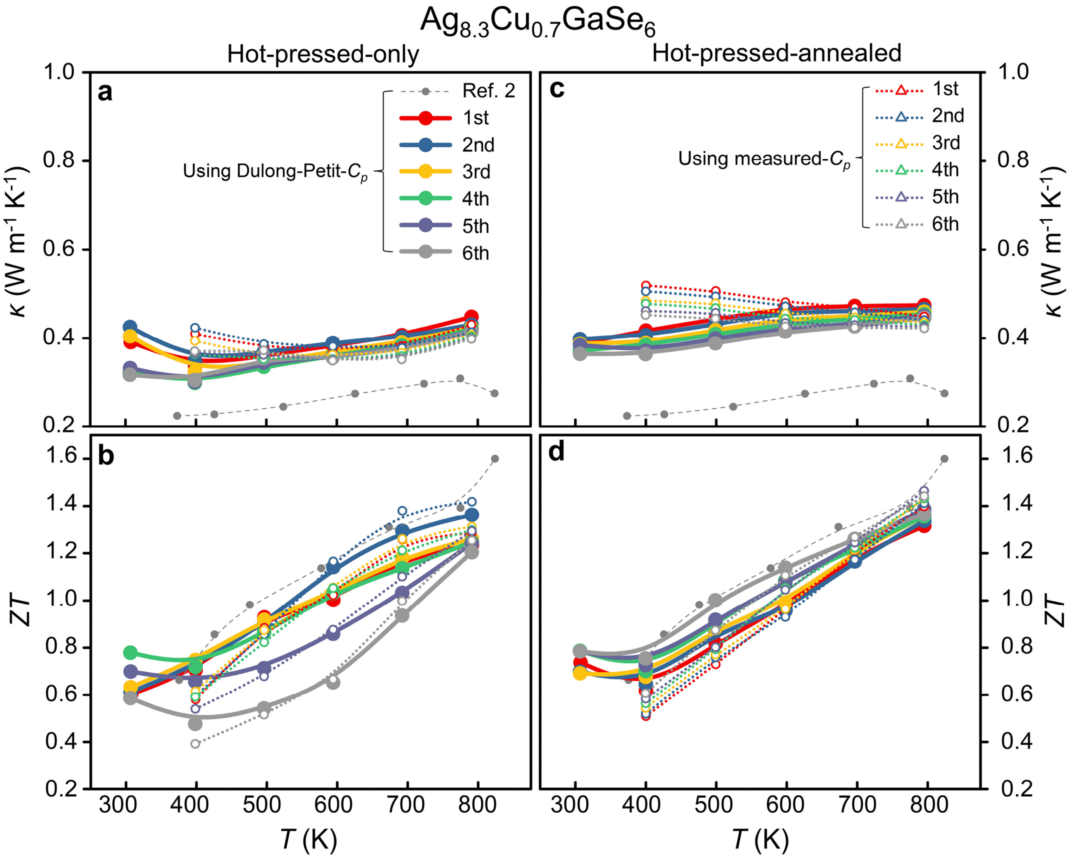

Supplementary Fig. 4. Temperature-dependent thermal conductivity (κ) and figure of merit (*ZT*) of $Ag_{8.3}Cu_{0.7}GaSe_6$: **a, b,** hot-pressed-only and **c, d,** hot-pressed-annealed samples. The κ and *ZT* calculated by both the measured-C_p and Dulong-Petit-C_p are presented. Those reported in ref. 2 that are calculated by the Dulong-Petit-C_p are also presented for comparison.

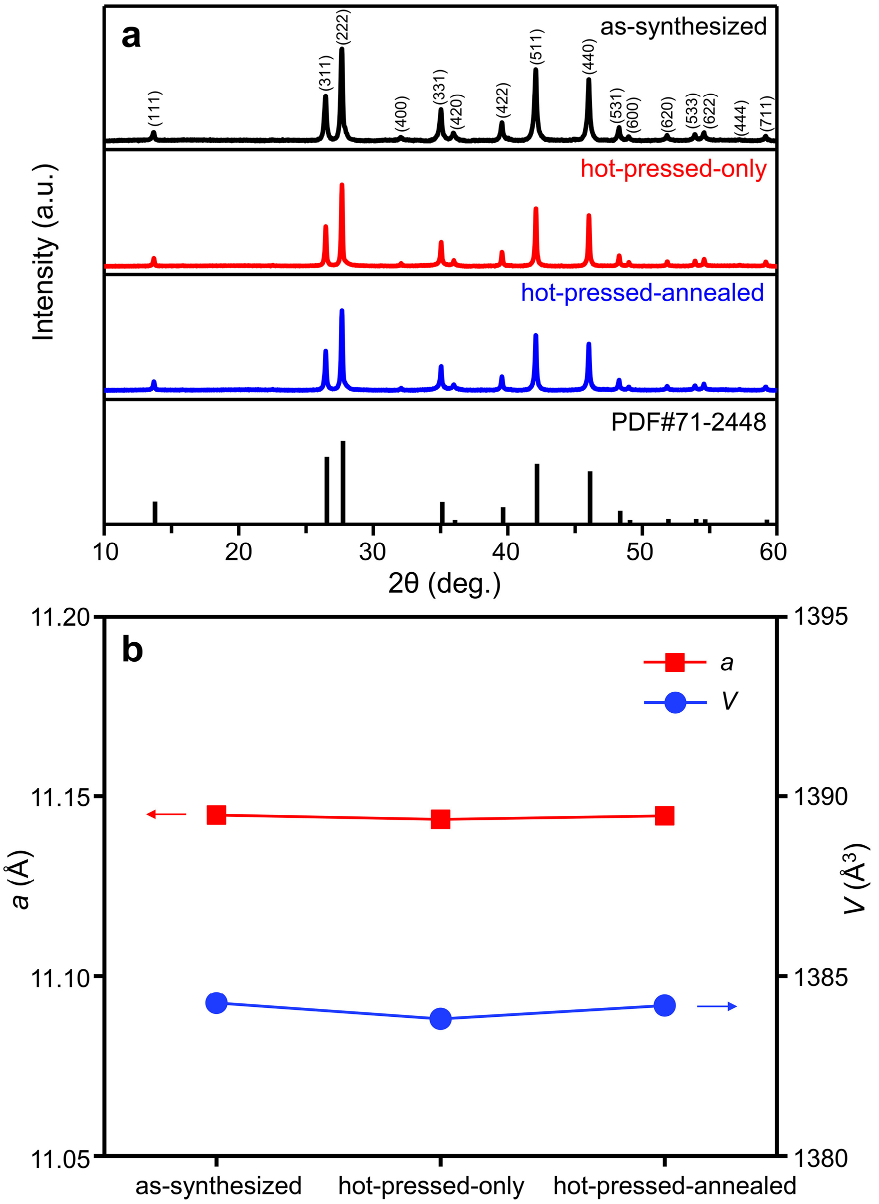

Supplementary Fig. 5. Powder XRD data and calculated cell parameters of all samples. **a**, Ag_9GaSe_6 powder X-ray diffraction patterns of the as-synthesized, hot-pressed-only, hot-pressed-annealed samples and the standard PDF#71-2448 (space group $F\bar{4}3m$). **b**, Comparison of the calculated unit cell parameter a and volume V.

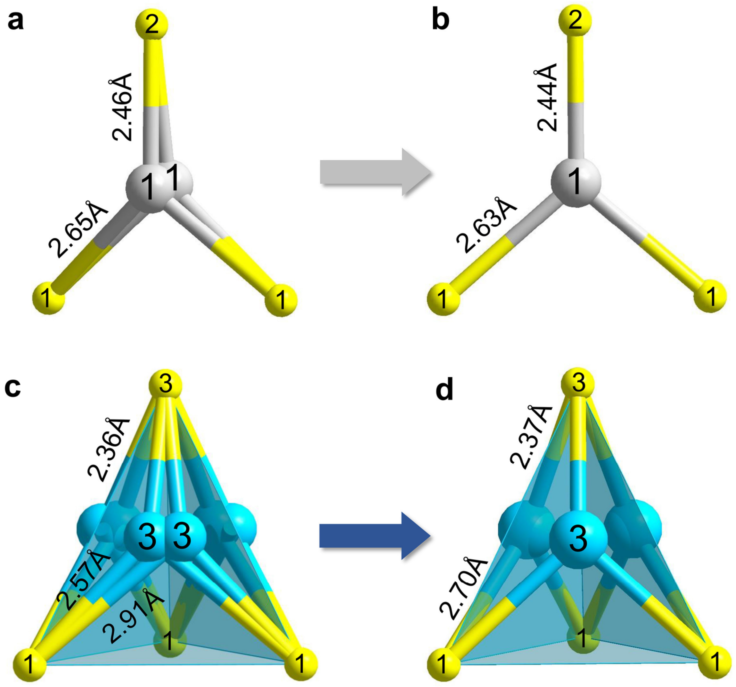

Supplementary Fig. 6. Local coordination of Ag1 and Ag3 atoms. The split **a**) Ag1 and **c**) Ag3 atoms in hot-pressed-annealed sample with the bond lengths marked. **b**, **d**, herein we treat these equivalent split Ag1 and Ag3 as a whole and the weighted average bond lengths are labeled.

Scanning electron microscopy for Ag_9GaSe_6 sample

SEM was perfomed to find out if the metal precipitation can be observed. As shown in Supplementary Fig. 7a and b, obvious heterogeneous particles and uneven surface are observed after measurement in the hot-pressed-only sample with nanoscale magnification, compared with the relatively clean and homogenous surface of the before-measured sample. Also, EDS mapping analysis in Supplementary Fig. 7e and f reveals the Ag content increases from ~ 52% (closed to the ratio in Ag_9GaSe_6) to ~ 63% after measurement. Similar increase of Ag content before and after property measurement is also reported by Luo et al. in ref. 2 (from ~ 52%/~ 55% to ~ 64%/~ 66%), in which this Ag-rich phenomenon is attributed to Ag ion migration under temperature difference during measurement.

Significantly, both before and after the test, the hot-pressed-annealed sample throughout shows homogenous, flat and clean cross section (Supplementary Fig. 7c and d), and the Ag content also hardly changes (from ~ 50% to ~ 51% in Supplementary Fig. 7g and h), indicating the positive effect of annealing on stabilizing the phase composition of Ag_9GaSe_6.

Thus, with the aid of SEM and EDS, we demonstrate annealing are benefit to eliminate the Ag migration induced Ag-rich phenomenon and maintain the phase uniformity and composition stability.

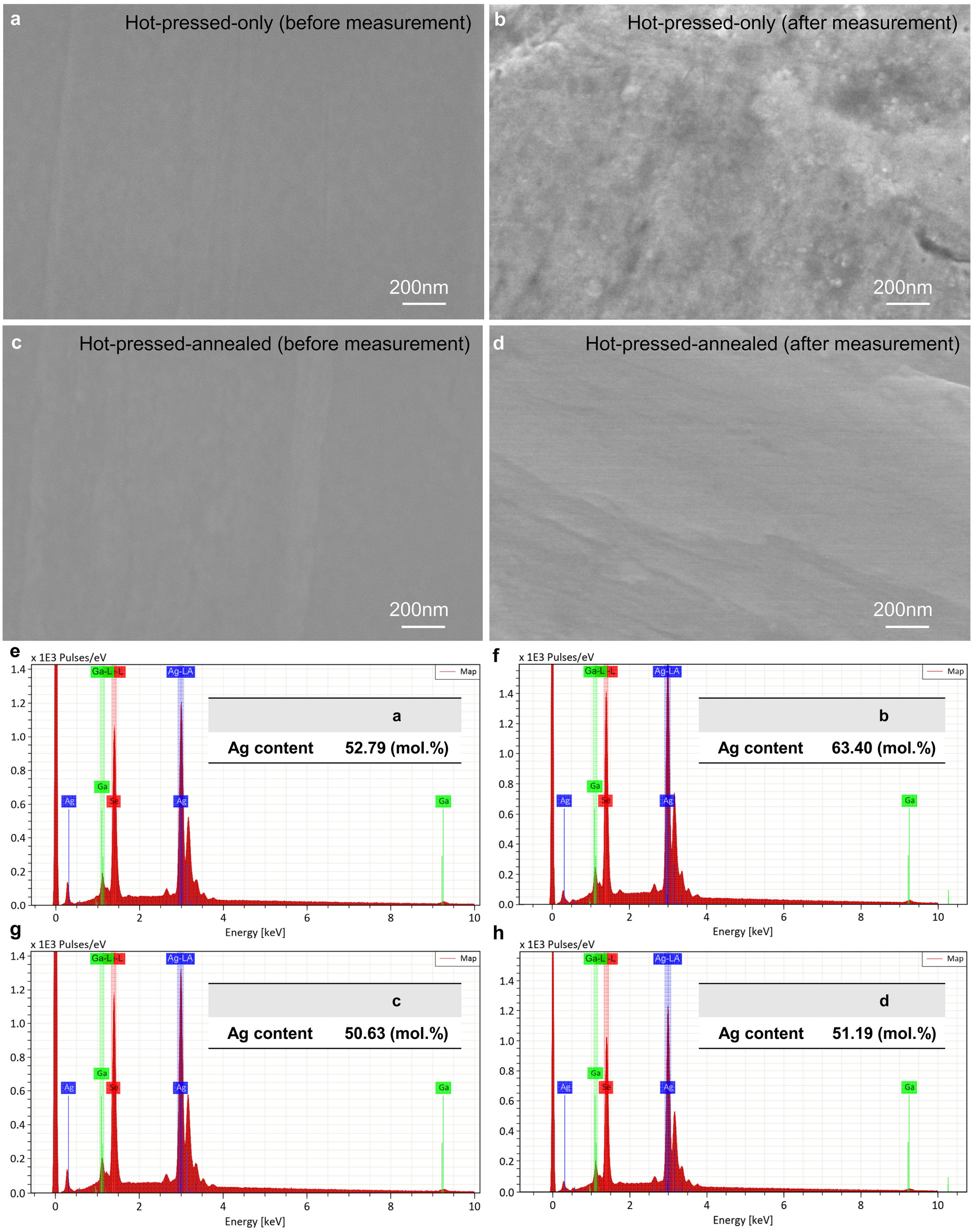

Supplementary Fig. 7. SEM images of samples before and after measurement. (**a**–**d**) SEM images of the cross section and (**e**–**h**) elemental distribution scanning spectra of the hot-pressed-only and hot-pressed-annealed Ag_9GaSe_6 samples before (**a**, **c**) and after (**b**, **d**) the 4-times TE-performance cycling tests, respectively. The Ag content for each sample is specially listed.

Ag migration observed under different pressure

To further establish the correlation between the Ag migration and the hot-press pressure, the hot-pressing procedures were carried out under different pressures (0–90 MPa), during which high quality single crystals were obtained. (Supplementary Tables 1–12) The 16 sets of single crystal diffraction data of such Ag_9GaSe_6 single crystals (Supplementary Tables 10–12) that were as-grown inside the ingots made under hot-pressing with different pressures (that was set in the range of 0–90 MPa, note that the 90 MPa is the up-limit of our hot-pressing facility. And another 18 sets single crystal diffraction data were collected on single crystals as-grown inside the ingots that were made under the standard hot-press pressure of 60 MPa, and 9 sets were collected on single crystals as-grown inside the ingots inside silica tubings that were sealed under vacuum with a residual pressure of less than 10^{-3} Pa (Supplementary Tables 1–9). These experimental data demonstrated when the hot-press-pressure is higher than 60 MPa, the Ag atoms migrate away from the 3-fold coordinated sites to the 4-fold coordinated sites. (Supplementary Fig. 8)

Below 60 MPa, all Ag atoms exhibit relatively constant occupancy as pressure changes, indicating almost no migration occurs. Higher than 60 MPa, the occupancy of Ag1 and Ag3 decreases and that of Ag4 and Ag5 increases, whereas that of Ag2 almost unchange. (Supplementary Fig. 8a) More interestingly, above 60 MPa, the decrease of the population of the 3-fold-coordinated Ag atoms equals to that of the 4-fold-coordinated Ag atoms, which clearly shows the Ag atoms migrates from the 3-fold-coordinated sites to the the 4-fold-coordinated sites. (Supplementary Fig. 8b)

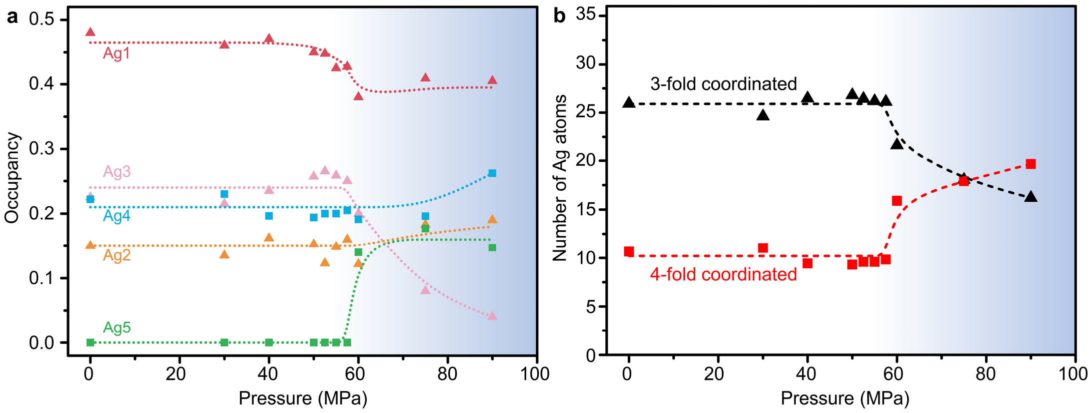

Supplementary Fig. 8. Pressure-dependent Ag occupancy and coordination number. Hot-press pressure-dependent **a**) average occupancy of each Ag atom and **b**) total numbers per unit cell of the 3-fold- (Ag1 + Ag2 + Ag3) and 4-fold-coordinated (Ag4 + Ag5) Ag atoms, respectively. Points are experimental data listed in Supplementary Tables 10–12 and the dotted line is the fitting curve.

Scanning electron microscopy for Ag_9GaSe_6 sample surface before and after annealing

We performed the scanning electron microscopy (SEM) to find out whether changes had occurred on polished Ag_9GaSe_6 bulk samples after 1d-annealing at 823K (Supplementary Fig. 9). Compared with the literature,[4] no obvious Se-volatilization-induced pores and metal precipitation were observed in our after-annealed samples and the scratches caused by sanding are still clearly visible, indicating the unaffected sample surface after annealing. The elemental distribution mappings on the right also proved that the composition is uniform and homogenous.

What' more, after annealing the unit cell parameters calculated by powder-XRD data merely change by a factor less than 0.09‰ in the *a* parameter (11.1436 vs. 11.1446 Å) and 0.3‰ in *V* (1383.80 vs. 1384.17 $Å^3$) (Supplementary Fig. 5b). These negligible changes also indicate the constant and stable phase composition during annealing process.

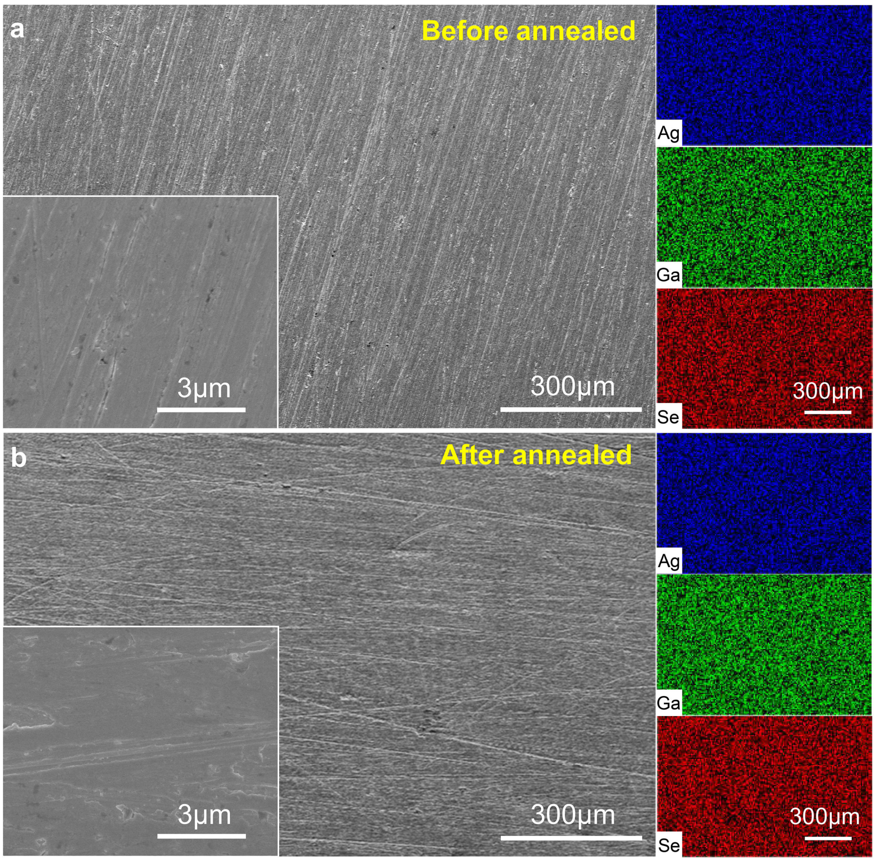

Supplementary Fig. 9. SEM images with the corresponding elemental distribution mappings of polished surfaces for Ag_9GaSe_6 bulk sample **a**), before and **b**), after being annealed for 1d at 823K. Insets show the magnified images for random-selected regions in (a) and (b).

Critical voltage determination

The critical voltage under constant temperature was measured in a reformed Netzsch DIL 402C equipment by monitoring the material's relative electrical resistance variation (R/R_0, where R_0 is the material's initial electrical resistance) before and after applying different electric currents on the sample. The details about the measurements can be found in Qiu et al.[5] The sample size was about 1.8 × 1.8 × 8.5 mm^3. The knee points found in the R/R_0 versus J scatter diagram corresponds to the critical current density (J_c). Based on the measured J_c, V_c under constant temperature can be obtained by the relation $V_c = J_c L/\sigma$, where L and σ are the length and electrical conductivity of the material, respectively. All the measurements were conducted in a chamber filled with high pure Argon.

We tested current density (J) dependence of relative electrical resistance variation (R/R_0) for hot-pressed-only and hot-pressed-annealed Ag_9GaSe_6, $Ag_{8.3}Cu_{0.7}GaSe_6$ and $Ag_9GaSe_{5.5}Te_{0.5}$ samples at 750 K. The R/R_0 of all hot-pressed-only samples exhibit almost linear decline as J increases (Supplementary Fig. 10a–c); while the hot-pressed-annealed Ag_9GaSe_6 and $Ag_9GaSe_{5.5}Te_{0.5}$ samples firstly show a plateau and then decrease sharply (Supplementary Fig. 10d and f), indicating the existence of critical voltage (V_c), though the J values corresponding to this inflection point are different, which is caused by different sample lengths (the sample lengths range from 8.33 to 8.58 mm)[5]. The appearance of critical voltage of these two samples represents the enhanced stability after annealing. The Cu-doped sample doesn't show a critical voltage after annealing (Supplementary Fig. 10e), probably because Cu atom is also liquid-like and even smaller than Ag, which aggravated the instability of the sample, so that the effect of annealing was not as obvious as the above two samples. Nevertheless, the annealing excels in the reproducibility of the TE properties of Cu-doped sample, as described in the main text.

Moreover, the critical voltages (V_c) at 750 K of annealed Ag_9GaSe_6 (0.05 V) and $Ag_9GaSe_{5.5}Te_{0.5}$ (0.07 V) are between Cu_2S (0.02 V) and Cu_2Se (0.11 V), indicating the comparable stability to these state-of-the-art Cu_2S/Se/Te-based liquid-like TE materials (Supplementary Fig. 11)[5].

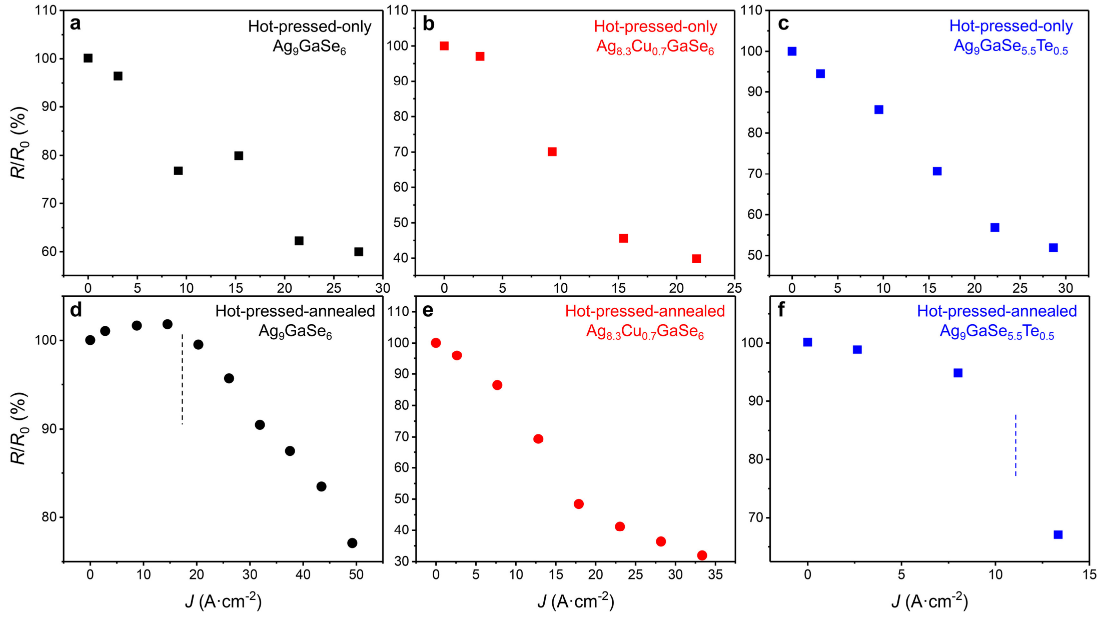

Supplementary Fig. 10. Current density dependence of relative electrical resistance variation (R/R_0) for all Ag_9GaSe_6-based samples. **a**, **b**, **c**, The hot-pressed-only Ag_9GaSe_6, $Ag_{8.3}Cu_{0.7}GaSe_6$ and $Ag_9GaSe_{5.5}Te_{0.5}$. **d**, **e**, **f**, The hot-pressed-annealed Ag_9GaSe_6, $Ag_{8.3}Cu_{0.7}GaSe_6$ and $Ag_9GaSe_{5.5}Te_{0.5}$. The vertical dotted line indicates the ending of the R/R_0 plateau and starting of the sharp decrease of the R/R_0 curve, indicating the existence of a critical voltage (V_c).

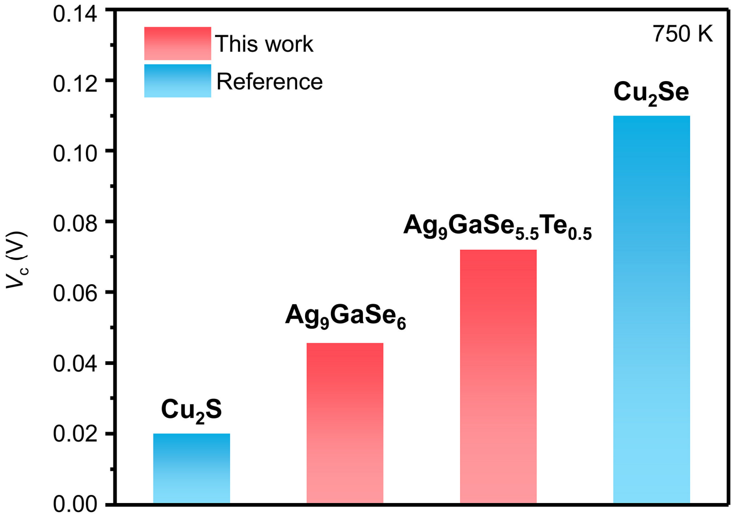

Supplementary Fig. 11. V_c of Ag_9GaSe_6-based and Cu_2Q-based materials. Experimentally determined V_c of Ag_9GaSe_6 and $Ag_9GaSe_{5.5}Te_{0.5}$ at 750 K and the reported values of Cu_2S and Cu_2Se[5] are also presented.

Supplementary Table 1. Crystallographic data and refinements of the as-synthesized single crystals #1– #9. The CCDC deposition numbers are 2170803 to 2170811.

	**#1**	**#2**	**#3**
**Empirical formula**	Ag_9GaSe_6	Ag_9GaSe_6	Ag_9GaSe_6
**formula weight**	1514.31	1514.31	1514.31
**crystal system**	Cubic	Cubic	Cubic
**crystal color**	Black	Black	Black
**space group**	$F\bar{4}3m$	$F\bar{4}3m$	$F\bar{4}3m$
$a = b = c$ **(Å)**	11.1244(2)	11.152(2)	11.150 (2)
$\alpha = \beta = \gamma$ **(°)**	90.00	90.00	90.00
V **(Å^3)**	1376.68(5)	1386.8(5)	1386.1(5)
Z	4	4	4
ρ_{cal} **(g/cm^3)**	7.306	7.253	7.257
μ **(mm^{-1})**	30.247	30.027	30.041
**GOOF on** F^2	1.049	1.072	1.070
R_1, wR_2 $(I > 2\sigma(I))$ [a]	0.0537, 0.1431	0.0587, 0.1449	0.0635, 0.1564
R_1, wR_2 **(all data)**	0.1073, 0.1796	0.0932, 0.1691	0.0976, 0.1821
**largest diff. peak and hole (e/Å^3)**	2.46/-1.13	2.61/-1.19	2.60/-1.32
	**#4**	**#5**	**#6**
**Empirical formula**	Ag_9GaSe_6	Ag_9GaSe_6	Ag_9GaSe_6
**formula weight**	1514.31	1514.31	1514.31
**crystal system**	Cubic	Cubic	Cubic
**crystal color**	Black	Black	Black
**space group**	$F\bar{4}3m$	$F\bar{4}3m$	$F\bar{4}3m$
$a = b = c$ **(Å)**	11.1211(2)	11.119(2)	11.163(3)
$\alpha = \beta = \gamma$ **(°)**	90.00	90.00	90.00
V **(Å^3)**	1375.45(4)	1374.7(5)	1390.9(6)
Z	4	4	4
ρ_{cal} **(g/cm^3)**	7.313	7.317	7.232
μ **(mm^{-1})**	30.274	30.290	29.938
**GOOF on** F^2	1.075	1.188	1.041
R_1, wR_2 $(I > 2\sigma(I))$ [a]	0.0568, 0.1673	0.0683, 0.1460	0.0543, 0.1245
R_1, wR_2 **(all data)**	0.0655, 0.1801	0.0919, 0.1597	0.0933, 0.1492
**largest diff. peak and hole (e/Å^3)**	2.97, −1.54	2.08, −1.85	2.45, −1.33
	**#7**	**#8**	**#9**
**Empirical formula**	Ag_9GaSe_6	Ag_9GaSe_6	Ag_9GaSe_6
**formula weight**	1514.31	1514.31	1514.31
**crystal system**	Cubic	Cubic	Cubic
**crystal color**	Black	Black	Black
**space group**	$F\bar{4}3m$	$F\bar{4}3m$	$F\bar{4}3m$
$a = b = c$ **(Å)**	11.127(2)	11.1291(6)	11.1233(5)
$\alpha = \beta = \gamma$ **(°)**	90.00	90.00	90.00
V **(Å^3)**	1377.5(5)	1378.42(13)	1376.26(11)
Z	4	4	4
ρ_{cal} **(g/cm^3)**	7.302	7.297	7.308
μ **(mm^{-1})**	30.229	30.209	30.256
**GOOF on** F^2	1.111	1.068	1.163
R_1, wR_2 $(I > 2\sigma(I))$ [a]	0.0532, 0.1195	0.0491, 0.1260	0.0564, 0.1369
R_1, wR_2 **(all data)**	0.0764, 0.1340	0.0868, 0.1529	0.0776, 0.1574
**largest diff. peak and hole (e/Å^3)**	1.69, −1.36	1.83, −0.99	2.56, −1.69

[a] $R_1 = \Sigma||F_o| - |F_c||/\Sigma|F_o|$, $wR_2 = [\Sigma w(F_o^2 - F_c^2)^2/\Sigma w(F_o^2)^2]^{1/2}$.

Supplementary Table 2. Crystallographic data and refinements of the hot-pressed-only single crystals #1 – #9. The CCDC deposition numbers are 2170812 to 2170820.

	#1	#2	#3
**Empirical formula**	Ag_9GaSe_6	Ag_9GaSe_6	Ag_9GaSe_6
**formula weight**	1514.31	1514.31	1514.31
**crystal system**	Cubic	Cubic	Cubic
**crystal color**	Black	Black	Black
**space group**	$F\bar{4}3m$	$F\bar{4}3m$	$F\bar{4}3m$
$a = b = c$ **(Å)**	11.108(2)	11.1179(5)	11.1166(8)
$\alpha = \beta = \gamma$ **(°)**	90.00	90.00	90.00
V **(Å^3)**	1370.6(5)	1374.25(11)	1373.78(17)
Z	4	4	4
ρ_{cal} **(g/cm^3)**	7.338	7.319	7.322
μ **(mm^{-1})**	30.380	30.300	30.311
**GOOF on** F^2	1.095	1.083	1.071
R_1, wR_2 ($I > 2\sigma(I)$)[a]	0.0652, 0.1694	0.0614, 0.1190	0.0769, 0.1857
R_1, wR_2 **(all data)**	0.0925, 0.1946	0.1047, 0.1422	0.1074, 0.2078
**largest diff. peak and hole (e/Å^3)**	2.13, −2.00	2.01, −1.85	2.74, −1.81
	**#4**	**#5**	**#6**
**Empirical formula**	Ag_9GaSe_6	Ag_9GaSe_6	Ag_9GaSe_6
**formula weight**	1514.31	1514.31	1514.31
**crystal system**	Cubic	Cubic	Cubic
**crystal color**	Black	Black	Black
**space group**	$F\bar{4}3m$	$F\bar{4}3m$	$F\bar{4}3m$
$a = b = c$ **(Å)**	11.1242(9)	11.1220(2)	11.1156(8)
$\alpha = \beta = \gamma$ **(°)**	90.00	90.00	90.00
V **(Å^3)**	1376.60(18)	1375.79(5)	1373.42(17)
Z	4	4	4
ρ_{cal} **(g/cm^3)**	7.307	7.311	7.324
μ **(mm^{-1})**	30.249	30.266	30.319
**GOOF on** F^2	1.090	1.162	1.180
R_1, wR_2 ($I > 2\sigma(I)$)[a]	0.0663, 0.1741	0.0573, 0.1490	0.0563, 0.1385
R_1, wR_2 **(all data)**	0.0952, 0.1953	0.0876, 0.1728	0.0999, 0.1690
**largest diff. peak and hole (e/Å^3)**	2.18, −1.53	2.62, −1.59	1.87, −1.78
	**#7**	**#8**	**#9**
**Empirical formula**	Ag_9GaSe_6	Ag_9GaSe_6	Ag_9GaSe_6
**formula weight**	1514.31	1514.31	1514.31
**crystal system**	Cubic	Cubic	Cubic
**crystal color**	Black	Black	Black
**space group**	$F\bar{4}3m$	$F\bar{4}3m$	$F\bar{4}3m$
$a = b = c$ **(Å)**	11.1187(10)	11.1151(5)	11.1237(8)
$\alpha = \beta = \gamma$ **(°)**	90.00	90.00	90.00
V **(Å^3)**	1374.6(2)	1373.22(11)	1376.41(17)
Z	4	4	4
ρ_{cal} **(g/cm^3)**	7.317	7.325	7.308
μ **(mm^{-1})**	30.293	30.323	30.253
**GOOF on** F^2	1.086	1.151	1.185
R_1, wR_2 ($I > 2\sigma(I)$)[a]	0.0673, 0.1794	0.0541, 0.1224	0.0520, 0.1193
R_1, wR_2 **(all data)**	0.1162, 0.2240	0.1190, 0.1554	0.0887, 0.1449
**largest diff. peak and hole (e/Å^3)**	2.03, −1.53	1.77, −1.42	2.55, −1.77

[a] $R_1 = \Sigma||F_o| - |F_c||/\Sigma|F_o|$, $wR_2 = [\Sigma w(F_o^2 - F_c^2)^2/\Sigma w(F_o^2)^2]^{1/2}$

Supplementary Table 3. Crystallographic data and refinements of the hot-pressed-annealed single crystals #1–#9. The CCDC deposition numbers are 2170821 to 2170829.

	**#1**	**#2**	**#3**
**Empirical formula**	Ag_9GaSe_6	Ag_9GaSe_6	Ag_9GaSe_6
**formula weight**	1514.31	1514.31	1514.31
**crystal system**	Cubic	Cubic	Cubic
**crystal color**	Black	Black	Black
**space group**	$F\bar{4}3m$	$F\bar{4}3m$	$F\bar{4}3m$
$a = b = c$ **(Å)**	11.1211(4)	11.1239(3)	11.1205(2)
$\alpha = \beta = \gamma$ **(°)**	90.00	90.00	90.00
V **(Å^3)**	1375.45(9)	1376.48(6)	1375.22(4)
Z	4	4	4
ρ_{cal} **(g/cm^3)**	7.313	7.307	7.314
μ **(mm^{-1})**	30.274	30.251	30.279
**GOOF on** F^2	1.047	1.075	1.028
R_1, wR_2 ($I > 2\sigma(I)$) [a]	0.0567, 0.1319	0.0514, 0.1137	0.0551, 0.1243
R_1, wR_2 **(all data)**	0.1174, 0.1688	0.0976, 0.1457	0.0950, 0.1480
**largest diff. peak and hole (e/Å^3)**	2.00, −1.46	1.72, −1.52	2.08, −1.58
	**#4**	**#5**	**#6**
**Empirical formula**	Ag_9GaSe_6	Ag_9GaSe_6	Ag_9GaSe_6
**formula weight**	1514.31	1514.31	1514.31
**crystal system**	Cubic	Cubic	Cubic
**crystal color**	Black	Black	Black
**space group**	$F\bar{4}3m$	$F\bar{4}3m$	$F\bar{4}3m$
$a = b = c$ **(Å)**	11.1382(15)	11.1428(19)	11.131(2)
$\alpha = \beta = \gamma$ **(°)**	90.00	90.00	90.00
V **(Å^3)**	1381.8(3)	1383.5(4)	1379.1(5)
Z	4	4	4
ρ_{cal} **(g/cm^3)**	7.279	7.270	7.293
μ **(mm^{-1})**	30.135	30.097	30.193
**GOOF on** F^2	0.970	1.063	1.119
R_1, wR_2 ($I > 2\sigma(I)$) [a]	0.0562, 0.1233	0.0428, 0.0989	0.0650, 0.1681
R_1, wR_2 **(all data)**	0.0949, 0.1471	0.0834, 0.1180	0.0833, 0.1807
**largest diff. peak and hole (e/Å^3)**	1.57, −2.03	2.39, −2.51	2.26, −1.32
	**#7**	**#8**	**#9**
**Empirical formula**	Ag_9GaSe_6	Ag_9GaSe_6	Ag_9GaSe_6
**formula weight**	1514.31	1514.31	1514.31
**crystal system**	Cubic	Cubic	Cubic
**crystal color**	Black	Black	Black
**space group**	$F\bar{4}3m$	$F\bar{4}3m$	$F\bar{4}3m$
$a = b = c$ **(Å)**	11.1174(7)	11.124(2)	11.1252(5)
$\alpha = \beta = \gamma$ **(°)**	90.00	90.00	90.00
V **(Å^3)**	1374.05(14)	1376.5(5)	1376.97(11)
Z	4	4	4
ρ_{cal} **(g/cm^3)**	7.320	7.307	7.305
μ **(mm^{-1})**	30.304	30.251	30.240
**GOOF on** F^2	1.079	1.067	1.097
R_1, wR_2 ($I > 2\sigma(I)$) [a]	0.0577, 0.1275	0.0669, 0.1643	0.0460, 0.1067
R_1, wR_2 **(all data)**	0.0859, 0.1441	0.1112, 0.1943	0.0941, 0.1327
**largest diff. peak and hole (e/Å^3)**	1.98, −2.94	2.37, −2.20	2.12, −2.17

[a] $R_1 = \Sigma||F_o| - |F_c||/\Sigma|F_o|$, $wR_2 = [\Sigma w(F_o^2 - F_c^2)^2/\Sigma w(F_o^2)^2]^{1/2}$

Supplementary Table 4. Atomic coordinates and equivalent isotropic displacement parameters and occupancies of the as-synthesized single crystals #1 – #9.

Atom site	*x*	*y*	*z*	*Occu.*	Ueq/Å^2	Wyck.
			**#1**			
Ag1	0.25000	0.25000	0.03250	0.494	0.114	24*g*
Ag2	0.29400	0.00000	0.50000	0.145	0.250	24*f*
Ag3	0.34810	0.15190	0.53800	0.250	0.200	48*h*
Ag4	0.32020	0.17980	0.48070	0.180	0.078	48*h*
Ga1	0.00000	0.00000	0.50000	1.000	0.027	4*b*
Se1	0.12336	0.12336	0.62336	1.000	0.034	16*e*
Se2	0.25000	0.25000	0.25000	1.000	0.072	4*c*
Se3	0.00000	0.50000	0.50000	1.000	0.100	4*a*
			**#2**			
Ag1	0.25000	0.25000	0.03330	0.458	0.095	24*g*
Ag2	0.29600	0.00000	0.50000	0.160	0.310	24*f*
Ag3	0.34720	0.15280	0.53700	0.240	0.188	48*h*
Ag4	0.31830	0.18170	0.47940	0.201	0.077	48*h*
Ga1	0.00000	0.00000	0.50000	1.000	0.019	4*b*
Se1	0.12336	0.12336	0.62336	1.000	0.026	16*e*
Se2	0.25000	0.25000	0.25000	1.000	0.063	4*c*
Se3	0.00000	0.50000	0.50000	1.000	0.093	4*a*
			**#3**			
Ag1	0.25000	0.25000	0.03450	0.423	0.087	24*g*
Ag2	0.29800	0.00000	0.50000	0.170	0.370	24*f*
Ag3	0.34740	0.15260	0.53500	0.240	0.190	48*h*
Ag4	0.31650	0.18350	0.47950	0.210	0.083	48*h*
Ga1	0.00000	0.00000	0.50000	1.000	0.022	4*b*
Se1	0.12347	0.12347	0.62347	1.000	0.029	16*e*
Se2	0.25000	0.25000	0.25000	1.000	0.066	4*c*
Se3	0.00000	0.50000	0.50000	1.000	0.096	4*a*
			**#4**			
Ag1	0.25000	0.25000	0.03410	0.427	0.087	24*g*
Ag2	0.29400	0.00000	0.50000	0.128	0.210	24*f*
Ag3	0.34580	0.15420	0.53100	0.267	0.190	48*h*
Ag4	0.31590	0.18410	0.47830	0.206	0.077	48*h*
Ga1	0.00000	0.00000	0.50000	1.000	0.020	4*b*
Se1	0.12350	0.12350	0.62350	1.000	0.026	16*e*
Se2	0.25000	0.25000	0.25000	1.000	0.064	4*c*
Se3	0.00000	0.50000	0.50000	1.000	0.098	4*a*
			**#5**			
Ag1	0.25000	0.25000	0.03310	0.464	0.106	24*g*
Ag2	0.29500	0.00000	0.50000	0.170	0.270	24*f*
Ag3	0.35250	0.14750	0.54600	0.210	0.151	48*h*

Ag4	0.32130	0.17870	0.48050	0.221	0.076	48*h*
Ga1	0.00000	0.00000	0.50000	1.000	0.021	4*b*
Se1	0.12348	0.12348	0.62348	1.000	0.029	16*e*
Se2	0.25000	0.25000	0.25000	1.000	0.063	4*c*
Se3	0.00000	0.50000	0.50000	1.000	0.096	4*a*
			**#6**			
Ag1	0.25000	0.25000	0.03300	0.478	0.103	24*g*
Ag2	0.29900	0.00000	0.50000	0.180	0.380	24*f*
Ag3	0.34950	0.15050	0.54100	0.223	0.167	48*h*
Ag4	0.31940	0.18060	0.47880	0.198	0.074	48*h*
Ga1	0.00000	0.00000	0.50000	1.000	0.022	4*b*
Se1	0.12355	0.12355	0.62355	1.000	0.030	16*e*
Se2	0.25000	0.25000	0.25000	1.000	0.067	4*c*
Se3	0.00000	0.50000	0.50000	1.000	0.095	4*a*
			**#7**			
Ag1	0.25000	0.25000	0.03260	0.499	0.111	24*g*
Ag2	0.29700	0.00000	0.50000	0.180	0.410	24*f*
Ag3	0.35290	0.14710	0.55000	0.187	0.143	48*h*
Ag4	0.32240	0.17660	0.48010	0.224	0.077	48*h*
Ga1	0.00000	0.00000	0.50000	1.000	0.024	4*b*
Se1	0.12354	0.12354	0.62354	1.000	0.030	16*e*
Se2	0.25000	0.25000	0.25000	1.000	0.066	4*c*
Se3	0.00000	0.50000	0.50000	1.000	0.094	4*a*
			**#8**			
Ag1	0.25000	0.25000	0.03220	0.482	0.109	24*g*
Ag2	0.29700	0.00000	0.50000	0.180	0.400	24*f*
Ag3	0.35190	0.14810	0.55000	0.195	0.161	48*h*
Ag4	0.32120	0.17880	0.47940	0.226	0.084	48*h*
Ga1	0.00000	0.00000	0.50000	1.000	0.030	4*b*
Se1	0.12343	0.12343	0.62343	1.000	0.036	16*e*
Se2	0.25000	0.25000	0.25000	1.000	0.074	4*c*
Se3	0.00000	0.50000	0.50000	1.000	0.103	4*a*
			**#9**			
Ag1	0.25000	0.25000	0.03340	0.460	0.096	24*g*
Ag2	0.29700	0.00000	0.50000	0.170	0.370	24*f*
Ag3	0.34740	0.15260	0.53400	0.240	0.187	48*h*
Ag4	0.31820	0.18180	0.47910	0.196	0.074	48*h*
Ga1	0.00000	0.00000	0.50000	1.000	0.021	4*b*
Se1	0.12341	0.12341	0.62341	1.000	0.029	16*e*
Se2	0.25000	0.25000	0.25000	1.000	0.066	4*c*
Se3	0.00000	0.50000	0.50000	1.000	0.096	4*a*

Supplementary Table 5. Atomic coordinates and equivalent isotropic displacement parameters and occupancies of the hot-pressed-only single crystals #1 – #9.

Atom site	*x*	*y*	*z*	*Occu.*	*U*eq/Å^2	Wyck.
			#1			
Ag1	0.25000	0.25000	−0.03450	0.388	0.083	24*g*
Ag2	0.00000	0.00000	−1.79500	0.210	0.340	24*f*
Ag3	0.16600	0.08600	0.08600	0.036	0.100	48*h*
Ag4	0.18630	0.18630	−0.02080	0.200	0.082	48*h*
Ag5	0.15650	0.15650	−0.02300	0.220	0.160	48*h*
Ga1	0.50000	0.00000	0.00000	1.000	0.021	4*b*
Se1	0.37659	−0.12341	−0.12341	1.000	0.027	16*e*
Se2	0.25000	0.25000	−0.25000	1.000	0.064	4*d*
Se3	0.00000	0.00000	0.00000	1.000	0.099	4*a*
			#2			
Ag1	0.25000	0.25000	−0.04280	0.249	0.070	24*g*
Ag2	0.00000	0.00000	−1.79200	0.134	0.260	24*f*
Ag3	0.15840	0.09610	0.09610	0.151	0.200	48*h*
Ag4	0.21600	0.21600	−0.02030	0.160	0.081	48*h*
Ag5	0.17100	0.17100	−0.01890	0.247	0.103	48*h*
Ga1	0.50000	0.00000	0.00000	1.000	0.022	4*b*
Se1	0.37654	−0.12346	−0.12354	1.000	0.029	16*e*
Se2	0.25000	0.25000	−0.25000	1.000	0.064	4*d*
Se3	0.00000	0.00000	0.00000	1.000	0.096	4*a*
			#3			
Ag1	0.25000	0.25000	−0.03800	0.234	0.088	24*g*
Ag2	0.00000	0.00000	−1.79000	0.128	0.260	24*f*
Ag3	0.16200	0.08700	0.08700	0.191	0.240	48*h*
Ag4	0.20900	0.20900	−0.01300	0.208	0.158	48*h*
Ag5	0.17290	0.17290	−0.02980	0.170	0.073	48*h*
Ga1	0.50000	0.00000	0.00000	1.000	0.019	4*b*
Se1	0.37644	−0.12356	−0.12356	1.000	0.028	16*e*
Se2	0.25000	0.25000	−0.25000	1.000	0.061	4*d*
Se3	0.00000	0.00000	0.00000	1.000	0.105	4*a*
			#4			
Ag1	0.25000	0.25000	−0.03470	0.400	0.081	24*g*
Ag2	0.00000	0.00000	−1.78800	0.064	0.120	24*f*
Ag3	0.15840	0.09360	0.09360	0.168	0.240	48*h*
Ag4	0.18820	0.18820	−0.02040	0.206	0.067	48*h*
Ag5	0.15960	0.15960	−0.02200	0.140	0.190	48*h*
Ga1	0.50000	0.00000	0.00000	1.000	0.021	4*b*
Se1	0.37653	−0.12347	−0.12347	1.000	0.028	16*e*
Se2	0.25000	0.25000	−0.25000	1.000	0.064	4*d*
Se3	0.00000	0.00000	0.00000	1.000	0.100	4*a*

			**#5**			
Ag1	0.25000	0.25000	−0.03290	0.461	0.095	24*g*
Ag2	0.00000	0.00000	−1.79300	0.139	0.250	24*f*
Ag3	0.16800	0.07200	0.07200	0.017	0.058	48*h*
Ag4	0.17980	0.17980	−0.02080	0.252	0.079	48*h*
Ag5	0.14800	0.14800	−0.05200	0.181	0.128	48*h*
Ga1	0.50000	0.00000	0.00000	1.000	0.022	4*b*
Se1	0.37659	−0.12341	−0.12341	1.000	0.028	16*e*
Se2	0.25000	0.25000	−0.25000	1.000	0.066	4*d*
Se3	0.00000	0.00000	0.00000	1.000	0.096	4*a*
			**#6**			
Ag1	0.25000	0.25000	−0.04130	0.227	0.066	24*g*
Ag2	0.00000	0.00000	−1.78700	0.200	0.310	24*f*
Ag3	0.16140	0.08990	0.08990	0.171	0.220	48*h*
Ag4	0.21200	0.21200	−0.02160	0.160	0.081	48*h*
Ag5	0.17060	0.10760	−0.01940	0.203	0.097	48*h*
Ga1	0.50000	0.00000	0.00000	1.000	0.023	4*b*
Se1	0.37667	−0.12333	−0.12333	1.000	0.029	16*e*
Se2	0.25000	0.25000	−0.25000	1.000	0.066	4*d*
Se3	0.00000	0.00000	0.00000	1.000	0.107	4*a*
			**#7**			
Ag1	0.25000	0.25000	−0.03380	0.438	0.099	24*g*
Ag2	0.00000	0.00000	−1.79400	0.173	0.288	24*f*
Ag3	0.16200	0.10100	0.10100	0.055	0.133	48*h*
Ag4	0.18280	0.18280	−0.02030	0.195	0.077	48*h*
Ag5	0.15390	0.15390	0.02900	0.190	0.150	48*h*
Ga1	0.50000	0.00000	0.00000	1.000	0.025	4*b*
Se1	0.37651	−0.12349	−0.12349	1.000	0.032	16*e*
Se2	0.25000	0.25000	−0.25000	1.000	0.069	4*d*
Se3	0.00000	0.00000	0.00000	1.000	0.106	4*a*
			**#8**			
Ag1	0.25000	0.25000	−0.04500	0.155	0.099	24*g*
Ag2	0.00000	0.00000	−1.79100	0.096	0.170	24*f*
Ag3	0.16180	0.09270	0.09270	0.190	0.250	48*h*
Ag4	0.22100	0.22100	−0.02000	0.262	0.149	48*h*
Ag5	0.17120	0.17120	−0.03080	0.173	0.079	48*h*
Ga1	0.50000	0.00000	0.00000	1.000	0.021	4*b*
Se1	0.37685	−0.12315	−0.12315	1.000	0.028	16*e*
Se2	0.25000	0.25000	−0.25000	1.000	0.062	4*d*
Se3	0.00000	0.00000	0.00000	1.000	0.101	4*a*
			**#9**			
Ag1	0.25000	0.25000	−0.03280	0.493	0.105	24*g*
Ag2	0.00000	0.00000	−1.79400	0.092	0.150	24*f*
Ag3	0.15710	0.09480	0.09480	0.118	0.180	48*h*

Ag4	0.18280	0.18280	−0.01990	0.176	0.060	48*h*
Ag5	0.15740	0.15740	−0.00300	0.160	0.200	48*h*
Ga1	0.50000	0.00000	0.00000	1.000	0.022	4*b*
Se1	0.37665	−0.12335	−0.12335	1.000	0.029	16*e*
Se2	0.25000	0.25000	−0.25000	1.000	0.066	4*d*
Se3	0.00000	0.00000	0.00000	1.000	0.097	4*a*

Supplementary Table 6. Atomic coordinates and equivalent isotropic displacement parameters and occupancies of the hot-pressed-annealed single crystals #1 – #9.

**Atom site**	***x***	***y***	***z***	***Occu.***	***U*eq/Å²**	**Wyck.**
			**#1**			
Ag1	0.23600	0.23600	−0.03060	0.235	0.088	48*h*
Ag2	0.00000	0.20900	0.00000	0.170	0.290	24*f*
Ag3	0.13500	0.15600	0.05000	0.102	0.164	96*i*
Ag4	0.17730	0.17730	−0.02120	0.224	0.078	48*h*
Ga1	0.00000	0.50000	0.00000	1.000	0.024	4*b*
Se1	0.12356	0.37644	0.12356	1.000	0.031	16*e*
Se2	0.25000	0.25000	−0.25000	1.000	0.067	4*d*
Se3	0.00000	0.00000	0.00000	1.000	0.104	4*a*
			**#2**			
Ag1	0.23500	0.23500	−0.03130	0.248	0.090	48*h*
Ag2	0.00000	0.20800	0.00000	0.062	0.110	24*f*
Ag3	0.13200	0.16800	0.02700	0.147	0.227	96*i*
Ag4	0.17890	0.17890	−0.02200	0.176	0.070	48*h*
Ga1	0.00000	0.50000	0.00000	1.000	0.024	4*b*
Se1	0.12337	0.37663	0.12337	1.000	0.030	16*e*
Se2	0.25000	0.25000	−0.25000	1.000	0.066	4*d*
Se3	0.00000	0.00000	0.00000	1.000	0.097	4*a*
			**#3**			
Ag1	0.23500	0.23500	−0.03150	0.249	0.088	48*h*
Ag2	0.00000	0.20700	0.00000	0.094	0.200	24*f*
Ag3	0.13500	0.16400	0.03400	0.132	0.218	96*i*
Ag4	0.17830	0.17830	−0.02210	0.189	0.077	48*h*
Ga1	0.00000	0.50000	0.00000	1.000	0.023	4*b*
Se1	0.12338	0.37662	0.12338	1.000	0.030	16*e*
Se2	0.25000	0.25000	−0.25000	1.000	0.066	4*d*
Se3	0.00000	0.00000	0.00000	1.000	0.097	4*a*
			**#4**			
Ag1	0.23230	0.23230	−0.03000	0.231	0.071	48*h*
Ag2	0.00000	0.20500	0.00000	0.117	0.190	24*f*
Ag3	0.13400	0.16200	0.03900	0.128	0.175	96*i*
Ag4	0.17770	0.17770	−0.02330	0.205	0.067	48*h*
Ga1	0.00000	0.50000	0.00000	1.000	0.020	4*b*
Se1	0.12339	0.37661	0.12339	1.000	0.027	16*e*
Se2	0.25000	0.25000	−0.25000	1.000	0.063	4*d*
Se3	0.00000	0.00000	0.00000	1.000	0.098	4*a*
			**#5**			
Ag1	0.23300	0.23300	−0.03090	0.259	0.090	48*h*
Ag2	0.00000	0.20700	0.00000	0.084	0.170	24*f*
Ag3	0.13500	0.16630	0.03100	0.140	0.223	96*i*

Ag4	0.17790	0.17790	−0.02170	0.170	0.073	48*h*
Ga1	0.00000	0.50000	0.00000	1.000	0.023	4*b*
Se1	0.12342	0.37658	0.12342	1.000	0.030	16*e*
Se2	0.25000	0.25000	−0.25000	1.000	0.065	4*d*
Se3	0.00000	0.00000	0.00000	1.000	0.097	4*a*
			**#6**			
Ag1	0.23380	0.23380	−0.03280	0.220	0.062	48*h*
Ag2	0.00000	0.20700	0.00000	0.068	0.132	24*f*
Ag3	0.13700	0.16400	0.02900	0.138	0.225	96*i*
Ag4	0.18100	0.18100	−0.02310	0.220	0.076	48*h*
Ga1	0.00000	0.50000	0.00000	1.000	0.021	4*b*
Se1	0.12342	0.37658	0.12342	1.000	0.028	16*e*
Se2	0.25000	0.25000	−0.25000	1.000	0.064	4*d*
Se3	0.00000	0.00000	0.00000	1.000	0.098	4*a*
			**#7**			
Ag1	0.23200	0.23200	−0.03070	0.238	0.083	48*h*
Ag2	0.00000	0.20500	0.00000	0.160	0.290	24*f*
Ag3	0.13600	0.16200	0.03800	0.121	0.180	96*i*
Ag4	0.17720	0.17720	−0.02320	0.190	0.071	48*h*
Ga1	0.00000	0.50000	0.00000	1.000	0.021	4*b*
Se1	0.12354	0.37646	0.12354	1.000	0.029	16*e*
Se2	0.25000	0.25000	−0.25000	1.000	0.061	4*d*
Se3	0.00000	0.00000	0.00000	1.000	0.104	4*a*
			**#8**			
Ag1	0.23170	0.23170	−0.03170	0.237	0.073	48*h*
Ag2	0.00000	0.20500	0.00000	0.123	0.220	24*f*
Ag3	0.13700	0.16300	0.03300	0.135	0.218	96*i*
Ag4	0.17810	0.17810	−0.02250	0.180	0.071	48*h*
Ga1	0.00000	0.50000	0.00000	1.000	0.021	4*b*
Se1	0.12342	0.37658	0.12342	1.000	0.028	16*e*
Se2	0.25000	0.25000	−0.25000	1.000	0.065	4*d*
Se3	0.00000	0.00000	0.00000	1.000	0.100	4*a*
			**#9**			
Ag1	0.23500	0.23500	−0.03170	0.239	0.080	48*h*
Ag2	0.00000	0.20600	0.00000	0.074	0.160	24*f*
Ag3	0.13300	0.16700	0.02900	0.142	0.228	96*i*
Ag4	0.17980	0.17980	−0.02170	0.190	0.071	48*h*
Ga1	0.00000	0.50000	0.00000	1.000	0.024	4*b*
Se1	0.12353	0.37647	0.12353	1.000	0.031	16*e*
Se2	0.25000	0.25000	−0.25000	1.000	0.066	4*d*
Se3	0.00000	0.00000	0.00000	1.000	0.097	4*a*

Supplementary Table 7. Anisotropic Displacement Parameters (Å^2) for of as-synthesized single crystals #1 – #9. The anisotropic displacement factor exponent takes the form: $-2\pi^2$ ($U_{11}h^2a^{*2}$ + $U_{22}k^2b^{*2}$ + $U_{33}l^2c^{*2}$ + $2U_{23}klb^*c^*$+ $2U_{13}hla^*c^*$ + $2U_{12}hka^*b^*$).

Atom site	U_{11}	U_{22}	U_{33}	U_{23}	U_{13}	U_{12}
			**#1**			
Ag1	0.151(9)	0.151(9)	0.0389(19)	0.000	0.000	0.097(9)
Ag2	0.049(10)	0.34(6)	0.34(6)	-0.11(5)	0.000	0.000
Ag3	0.127(9)	0.127(9)	0.34(4)	-0.072(14)	0.072(14)	0.032(10)
Ag4	0.085(5)	0.085(5)	0.065(6)	0.022(4)	-0.022(4)	-0.020(6)
Ga1	0.0267(8)	0.0267(8)	0.0267(8)	0.000	0.000	0.000
Se1	0.0339(5)	0.0339(5)	0.0339(5)	-0.0048(3)	-0.0048(3)	-0.0048(3)
Se2	0.0723(17)	0.0723(17)	0.0723(17)	0.000	0.000	0.000
Se3	0.100(3)	0.100(3)	0.100(3)	0.000	0.000	0.000
			**#2**			
Ag1	0.127(8)	0.127(8)	0.0306(18)	0.000	0.000	0.074(8)
Ag2	0.055(12)	0.43(10)	0.43(10)	-0.19(8)	0.000	0.000
Ag3	0.122(9)	0.122(9)	0.32(4)	-0.075(13)	0.075(13)	0.033(11)
Ag4	0.088(6)	0.088(6)	0.055(5)	0.026(4)	-0.026(4)	-0.030(6)
Ga1	0.0189(7)	0.0189(7)	0.0189(7)	0.000	0.000	0.000
Se1	0.0262(4)	0.0262(4)	0.0262(4)	-0.0046(3)	-0.0046(3)	-0.0046(3)
Se2	0.0629(16)	0.0629(16)	0.0629(16)	0.000	0.000	0.000
Se3	0.093(3)	0.093(3)	0.093(3)	0.000	0.000	0.000
			**#3**			
Ag1	0.115(7)	0.115(7)	0.032(2)	0.000	0.000	0.057(8)
Ag2	0.058(15)	0.53(15)	0.53(15)	-0.17(11)	0.000	0.000
Ag3	0.127(13)	0.127(13)	0.31(4)	-0.076(14)	0.076(14)	0.036(14)
Ag4	0.099(7)	0.099(7)	0.052(6)	0.023(5)	-0.023(5)	-0.039(8)
Ga1	0.0223(8)	0.0223(8)	0.0223(8)	0.000	0.000	0.000
Se1	0.0290(5)	0.0290(5)	0.0290(5)	-0.0045(3)	-0.0045(3)	-0.0045(3)
Se2	0.0663(19)	0.0663(19)	0.0663(19)	0.000	0.000	0.000
Se3	0.096(3)	0.096(3)	0.096(3)	0.000	0.000	0.000
			**#4**			
Ag1	0.116(7)	0.116(7)	0.0300(16)	0.000	0.000	0.059(7)
Ag2	0.053(12)	0.29(7)	0.29(7)	-0.12(6)	0.000	0.000
Ag3	0.130(8)	0.130(8)	0.31(3)	-0.077(11)	0.077(11)	0.031(10)
Ag4	0.092(6)	0.092(6)	0.046(4)	0.020(3)	-0.020(3)	-0.039(6)
Ga1	0.0203(5)	0.0203(5)	0.0203(5)	0.000	0.000	0.000
Se1	0.0262(4)	0.0262(4)	0.0262(4)	-0.0043(2)	-0.0043(2)	-0.0043(2)
Se2	0.0641(14)	0.0641(14)	0.0641(14)	0.000	0.000	0.000
Se3	0.098(3)	0.098(3)	0.098(3)	0.000	0.000	0.000
			**#5**			
Ag1	0.142(10)	0.142(10)	0.034(2)	0.000	0.000	0.089(10)
Ag2	0.076(19)	0.37(6)	0.37(6)	-0.14(5)	0.000	0.000

Ag3	0.132(13)	0.132(13)	0.19(3)	-0.035(11)	0.035(11)	0.066(13)
Ag4	0.083(5)	0.083(5)	0.060(6)	0.019(4)	-0.019(4)	-0.009(5)
Ga1	0.0209(9)	0.0209(9)	0.0209(9)	0.000	0.000	0.000
Se1	0.0285(5)	0.0285(5)	0.0285(5)	-0.0049(4)	-0.0049(4)	-0.0049(4)
Se2	0.0628(19)	0.0628(19)	0.0628(19)	0.000	0.000	0.000
Se3	0.096(4)	0.096(4)	0.096(4)	0.000	0.000	0.000
			**#6**			
Ag1	0.136(8)	0.136(8)	0.039(2)	0.000	0.000	0.080(8)
Ag2	0.059(12)	0.54(11)	0.54(11)	-0.17(8)	0.000	0.000
Ag3	0.119(11)	0.119(11)	0.26(4)	-0.060(12)	0.060(12)	0.036(11)
Ag4	0.084(5)	0.084(5)	0.052(5)	0.021(3)	-0.021(3)	-0.016(5)
Ga1	0.0222(7)	0.0222(7)	0.0222(7)	0.000	0.000	0.000
Se1	0.0298(4)	0.0298(4)	0.0298(4)	-0.0050(3)	-0.0050(3)	-0.0050(3)
Se2	0.0671(16)	0.0671(16)	0.0671(16)	0.000	0.000	0.000
Se3	0.095(3)	0.095(3)	0.095(3)	0.000	0.000	0.000
			**#7**			
Ag1	0.147(9)	0.147(9)	0.039(2)	0.000	0.000	0.093(9)
Ag2	0.058(13)	0.58(11)	0.58(11)	-0.25(9)	0.000	0.000
Ag3	0.117(11)	0.117(11)	0.19(3)	-0.037(9)	0.037(9)	0.049(10)
Ag4	0.084(4)	0.084(4)	0.063(5)	0.018(3)	-0.018(3)	-0.009(4)
Ga1	0.0240(8)	0.0240(8)	0.0240(8)	0.000	0.000	0.000
Se1	0.0303(4)	0.0303(4)	0.0303(4)	-0.0052(3)	-0.0052(3)	-0.0052(3)
Se2	0.0661(17)	0.0661(17)	0.0661(17)	0.000	0.000	0.000
Se3	0.094(3)	0.094(3)	0.094(3)	0.000	0.000	0.000
			**#8**			
Ag1	0.142(8)	0.142(8)	0.044(2)	0.000	0.000	0.083(9)
Ag2	0.055(12)	0.57(11)	0.57(11)	-0.26(8)	0.000	0.000
Ag3	0.122(11)	0.122(11)	0.24(4)	-0.044(12)	0.044(12)	0.046(10)
Ag4	0.091(5)	0.091(5)	0.071(6)	0.020(3)	-0.020(3)	-0.012(5)
Ga1	0.0297(9)	0.0297(9)	0.0297(9)	0.000	0.000	0.000
Se1	0.0363(5)	0.0363(5)	0.0363(5)	-0.0053(3)	-0.0053(3)	-0.0053(3)
Se2	0.0736(18)	0.0736(18)	0.0736(18)	0.000	0.000	0.000
Se3	0.103(3)	0.103(3)	0.103(3)	0.000	0.000	0.000
			**#9**			
Ag1	0.127(7)	0.127(7)	0.0345(19)	0.000	0.000	0.072(8)
Ag2	0.063(14)	0.53(13)	0.53(13)	-0.21(10)	0.000	0.000
Ag3	0.125(11)	0.125(11)	0.31(4)	-0.073(13)	0.073(13)	0.034(11)
Ag4	0.085(5)	0.085(5)	0.052(5)	0.022(4)	-0.022(4)	-0.026(6)
Ga1	0.0214(6)	0.0214(6)	0.0214(6)	0.000	0.000	0.000
Se1	0.0285(4)	0.0285(4)	0.0285(4)	-0.0051(3)	-0.0051(3)	-0.0051(3)
Se2	0.0664(16)	0.0664(16)	0.0664(16)	0.000	0.000	0.000
Se3	0.096(3)	0.096(3)	0.096(3)	0.000	0.000	0.000

Supplementary Table 8. Anisotropic Displacement Parameters (Å^2) for of hot-pressed-only single crystals #1 – #9. The anisotropic displacement factor exponent takes the form: $-2\pi^2$ ($U_{11}h^2a^{*2}$ + $U_{22}k^2b^{*2}$ + $U_{33}l^2c^{*2}$ + $2U_{23}klb^*c^*$+ $2U_{13}hla^*c^*$ + $2U_{12}hka^*b^*$).

Atom site	U_{11}	U_{22}	U_{33}	U_{23}	U_{13}	U_{12}
			**#1**			
Ag1	0.110(7)	0.110(7)	0.028(2)	0.000	0.000	0.052(8)
Ag2	0.47(12)	0.47(12)	0.080(19)	0.000	0.000	-0.16(9)
Ag3	0.08(3)	0.10(3)	0.10(3)	-0.005(19)	0.000(13)	0.000(13)
Ag4	0.098(8)	0.098(8)	0.049(5)	0.026(5)	0.026(5)	0.045(9)
Ag5	0.105(12)	0.105(12)	0.27(5)	-0.067(16)	-0.067(16)	-0.022(13)
Ga1	0.0208(7)	0.0208(7)	0.0208(7)	0.000	0.000	0.000
Se1	0.0273(4)	0.0273(4)	0.0273(4)	-0.0050(3)	-0.0050(3)	-0.0050(3)
Se2	0.0640(17)	0.0640(17)	0.0640(17)	0.000	0.000	0.000
Se3	0.099(4)	0.099(4)	0.099(4)	0.000	0.000	0.000
			**#2**			
Ag1	0.098(7)	0.098(7)	0.014(4)	0.000	0.000	0.015(9)
Ag2	0.37(6)	0.37(6)	0.044(14)	0.000	0.000	-0.19(5)
Ag3	0.052(9)	0.27(3)	0.27(3)	-0.16(3)	-0.023(7)	-0.023(7)
Ag4	0.11(2)	0.11(2)	0.021(5)	0.014(5)	0.014(5)	0.09(2)
Ag5	0.102(6)	0.102(6)	0.103(14)	0.004(4)	0.004(4)	-0.012(9)
Ga1	0.0221(8)	0.0221(8)	0.0221(8)	0.000	0.000	0.000
Se1	0.0290(4)	0.0290(4)	0.0290(4)	-0.0047(3)	-0.0047(3)	-0.0047(3)
Se2	0.0636(17)	0.0636(17)	0.0636(17)	0.000	0.000	0.000
Se3	0.096(3)	0.096(3)	0.096(3)	0.000	0.000	0.000
			**#3**			
Ag1	0.121(15)	0.121(15)	0.021(4)	0.000	0.000	0.024(15)
Ag2	0.37(8)	0.37(8)	0.048(15)	0.000	0.000	-0.30(9)
Ag3	0.069(11)	0.32(3)	0.32(3)	-0.17(3)	-0.045(11)	-0.045(11)
Ag4	0.20(3)	0.20(3)	0.076(14)	-0.080(17)	-0.080(17)	0.16(3)
Ag5	0.097(8)	0.097(8)	0.026(5)	0.001(3)	0.001(3)	-0.016(9)
Ga1	0.0193(8)	0.0193(8)	0.0193(8)	0.000	0.000	0.000
Se1	0.0277(5)	0.0277(5)	0.0277(5)	-0.0042(3)	-0.0042(3)	-0.0042(3)
Se2	0.0609(18)	0.0609(18)	0.0609(18)	0.000	0.000	0.000
Se3	0.105(5)	0.105(5)	0.105(5)	0.000	0.000	0.000
			**#4**			
Ag1	0.106(6)	0.106(6)	0.030(2)	0.000	0.000	0.038(8)
Ag2	0.16(4)	0.16(4)	0.025(13)	0.000	0.000	-0.12(4)
Ag3	0.049(8)	0.34(6)	0.34(6)	-0.22(6)	-0.022(8)	-0.022(8)
Ag4	0.077(7)	0.077(7)	0.047(5)	0.010(4)	0.010(4)	0.032(7)
Ag5	0.21(4)	0.21(4)	0.15(4)	0.007(10)	0.007(10)	-0.15(4)
Ga1	0.0209(8)	0.0209(8)	0.0209(8)	0.000	0.000	0.000
Se1	0.0275(5)	0.0275(5)	0.0275(5)	-0.0044(3)	-0.0044(3)	-0.0044(3)
Se2	0.0636(18)	0.0636(18)	0.0636(18)	0.000	0.000	0.000

Se3	0.100(4)	0.100(4)	0.100(4)	0.000	0.000	0.000
			**#5**			
Ag1	0.123(7)	0.123(7)	0.038(2)	0.000	0.000	0.069(8)
Ag2	0.36(6)	0.36(6)	0.035(10)	0.000	0.000	-0.16(5)
Ag3	0.054(18)	0.059(14)	0.059(14)	-0.002(10)	-0.004(7)	-0.004(7)
Ag4	0.087(4)	0.087(4)	0.065(5)	0.016(3)	0.016(3)	0.018(5)
Ag5	0.100(10)	0.100(10)	0.19(3)	-0.026(8)	-0.026(8)	-0.042(11)
Ga1	0.0216(7)	0.0216(7)	0.0216(7)	0.000	0.000	0.000
Se1	0.0277(4)	0.0277(4)	0.0277(4)	-0.0049(3)	-0.0049(3)	-0.0049(3)
Se2	0.0659(18)	0.0659(18)	0.0659(18)	0.000	0.000	0.000
Se3	0.096(3)	0.096(3)	0.096(3)	0.000	0.000	0.000
			**#6**			
Ag1	0.092(7)	0.092(7)	0.016(3)	0.000	0.000	0.004(9)
Ag2	0.43(8)	0.43(8)	0.076(14)	0.000	0.000	-0.23(7)
Ag3	0.054(8)	0.30(3)	0.30(3)	-0.16(3)	-0.033(9)	-0.033(9)
Ag4	0.11(2)	0.11(2)	0.025(4)	0.010(5)	0.010(5)	0.082(19)
Ag5	0.096(7)	0.096(7)	0.098(16)	0.011(4)	0.011(4)	-0.013(10)
Ga1	0.0225(7)	0.0225(7)	0.0225(7)	0.000	0.000	0.000
Se1	0.0293(4)	0.0293(4)	0.0293(4)	-0.0043(3)	-0.0043(3)	-0.0043(3)
Se2	0.0657(16)	0.0657(16)	0.0657(16)	0.000	0.000	0.000
Se3	0.107(4)	0.107(4)	0.107(4)	0.000	0.000	0.000
			**#7**			
Ag1	0.130(6)	0.130(6)	0.037(2)	0.000	0.000	0.070(7)
Ag2	0.289(16)	0.289(16)	0.29(2)	0.000	0.000	-0.002(10)
Ag3	0.13(2)	0.133(16)	0.133(16)	-0.004(10)	0.001(7)	0.001(7)
Ag4	0.089(6)	0.089(6)	0.051(5)	0.020(4)	0.020(4)	0.024(6)
Ag5	0.111(11)	0.111(11)	0.23(5)	-0.048(16)	-0.048(16)	-0.037(13)
Ga1	0.0254(7)	0.0254(7)	0.0254(7)	0.000	0.000	0.000
Se1	0.0318(4)	0.0318(4)	0.0318(4)	-0.0044(3)	-0.0044(3)	-0.0044(3)
Se2	0.0695(17)	0.0695(17)	0.0695(17)	0.000	0.000	0.000
Se3	0.106(4)	0.106(4)	0.106(4)	0.000	0.000	0.000
			**#8**			
Ag1	0.140(18)	0.140(18)	0.017(6)	0.000	0.000	-0.02(2)
Ag2	0.24(3)	0.24(3)	0.037(12)	0.000	0.000	-0.11(3)
Ag3	0.061(8)	0.34(3)	0.34(3)	-0.22(3)	-0.030(7)	-0.030(7)
Ag4	0.20(3)	0.20(3)	0.051(6)	-0.059(11)	-0.059(11)	0.16(3)
Ag5	0.099(6)	0.099(6)	0.040(7)	0.003(4)	0.003(4)	-0.017(8)
Ga1	0.0214(7)	0.0214(7)	0.0214(7)	0.000	0.000	0.000
Se1	0.0284(4)	0.0284(4)	0.0284(4)	-0.0046(3)	-0.0046(3)	-0.0046(3)
Se2	0.0622(15)	0.0622(15)	0.0622(15)	0.000	0.000	0.000
Se3	0.101(3)	0.101(3)	0.101(3)	0.000	0.000	0.000
			**#9**			
Ag1	0.141(8)	0.141(8)	0.0347(18)	0.000	0.000	0.082(9)
Ag2	0.20(3)	0.20(3)	0.036(11)	0.000	0.000	-0.09(3)

Ag3	0.043(8)	0.24(4)	0.24(4)	-0.12(3)	-0.022(8)	-0.022(8)
Ag4	0.065(5)	0.065(5)	0.048(5)	0.013(3)	0.013(3)	0.012(5)
Ag5	0.16(2)	0.16(2)	0.27(5)	0.004(11)	0.004(11)	-0.12(3)
Ga1	0.0219(7)	0.0219(7)	0.0219(7)	0.000	0.000	0.000
Se1	0.0285(4)	0.0285(4)	0.0285(4)	-0.0052(3)	-0.0052(3)	-0.0052(3)
Se2	0.0659(16)	0.0659(16)	0.0659(16)	0.000	0.000	0.000
Se3	0.097(3)	0.097(3)	0.097(3)	0.000	0.000	0.000

Supplementary Table 9. Anisotropic Displacement Parameters (Å^2) for the hot-pressed-annealed single crystals #1 – #9. The anisotropic displacement factor exponent takes the form: $-2\pi^2$ ($U_{11}h^2a^{*2} + U_{22}k^2b^{*2} + U_{33}l^2c^{*2} + 2U_{23}klb^*c^* + 2U_{13}hla^*c^* + 2U_{12}hka^*b^*$).

Atom site	U_{11}	U_{22}	U_{33}	U_{23}	U_{13}	U_{12}
			**#1**			
Ag1	0.11(3)	0.11(3)	0.043(3)	-0.014(11)	-0.014(11)	0.06(3)
Ag2	0.41(7)	0.051(12)	0.41(7)	0.000	-0.23(6)	0.000
Ag3	0.21(4)	0.075(13)	0.21(4)	-0.042(18)	-0.02(4)	-0.059(17)
Ag4	0.085(5)	0.085(5)	0.063(5)	0.012(4)	0.012(4)	0.005(5)
Ga1	0.0243(7)	0.0243(7)	0.0243(7)	0.000	0.000	0.000
Se1	0.0313(5)	0.0313(5)	0.0313(5)	0.0048(3)	-0.0048(3)	0.0048(3)
Se2	0.0665(16)	0.0665(16)	0.0665(16)	0.000	0.000	0.000
Se3	0.104(3)	0.104(3)	0.104(3)	0.000	0.000	0.000
			**#2**			
Ag1	0.11(3)	0.11(3)	0.038(3)	-0.009(10)	-0.009(10)	0.06(3)
Ag2	0.16(3)	0.017(13)	0.16(3)	0.000	-0.07(3)	0.000
Ag3	0.21(2)	0.111(12)	0.36(3)	-0.148(16)	0.02(3)	-0.063(15)
Ag4	0.077(6)	0.077(6)	0.057(6)	0.024(5)	0.024(5)	0.015(7)
Ga1	0.0235(8)	0.0235(8)	0.0235(8)	0.000	0.000	0.000
Se1	0.0305(5)	0.0305(5)	0.0305(5)	0.0049(3)	-0.0049(3)	0.0049(3)
Se2	0.0655(18)	0.0655(18)	0.0655(18)	0.000	0.000	0.000
Se3	0.097(3)	0.097(3)	0.097(3)	0.000	0.000	0.000
			**#3**			
Ag1	0.11(3)	0.11(3)	0.039(2)	-0.011(8)	-0.011(8)	0.05(3)
Ag2	0.28(6)	0.040(12)	0.28(6)	0.000	-0.16(5)	0.000
Ag3	0.19(2)	0.103(12)	0.36(3)	-0.134(17)	-0.01(3)	-0.048(15)
Ag4	0.085(5)	0.085(5)	0.060(5)	0.025(5)	0.025(5)	0.022(6)
Ga1	0.0228(6)	0.0228(6)	0.0228(6)	0.000	0.000	0.000
Se1	0.0302(4)	0.0302(4)	0.0302(4)	0.0047(3)	-0.0047(3)	0.0047(3)
Se2	0.0660(15)	0.0660(15)	0.0660(15)	0.000	0.000	0.000
Se3	0.097(3)	0.097(3)	0.097(3)	0.000	0.000	0.000
			**#4**			
Ag1	0.089(14)	0.089(14)	0.035(2)	-0.014(5)	-0.014(5)	0.040(14)
Ag2	0.27(5)	0.033(9)	0.27(5)	0.000	-0.12(4)	0.000
Ag3	0.20(3)	0.091(13)	0.23(3)	-0.073(15)	0.00(3)	-0.064(18)
Ag4	0.076(4)	0.076(4)	0.050(5)	0.011(3)	0.011(3)	0.004(5)
Ga1	0.0203(7)	0.0203(7)	0.0203(7)	0.000	0.000	0.000
Se1	0.0273(4)	0.0273(4)	0.0273(4)	0.0047(3)	-0.0047(3)	0.0047(3)
Se2	0.0628(15)	0.0628(15)	0.0628(15)	0.000	0.000	0.000
Se3	0.098(3)	0.098(3)	0.098(3)	0.000	0.000	0.000
			**#5**			
Ag1	0.12(2)	0.12(2)	0.040(2)	-0.012(7)	-0.012(7)	0.06(2)

Ag2	0.23(4)	0.046(12)	0.23(4)	0.000	-0.10(3)	0.000
Ag3	0.20(2)	0.105(10)	0.36(3)	-0.132(14)	-0.02(3)	-0.050(14)
Ag4	0.077(5)	0.077(5)	0.065(6)	0.021(5)	0.021(5)	0.012(6)
Ga1	0.0231(6)	0.0231(6)	0.0231(6)	0.000	0.000	0.000
Se1	0.0304(4)	0.0304(4)	0.0304(4)	0.0050(3)	-0.0050(3)	0.0050(3)
Se2	0.0654(13)	0.0654(13)	0.0654(13)	0.000	0.000	0.000
Se3	0.097(3)	0.097(3)	0.097(3)	0.000	0.000	0.000
			**#6**			
Ag1	0.077(12)	0.077(12)	0.033(2)	-0.006(4)	-0.006(4)	0.024(12)
Ag2	0.134(15)	0.129(19)	0.134(15)	0.000	-0.002(10)	0.000
Ag3	0.20(3)	0.121(15)	0.35(3)	-0.15(2)	0.03(3)	-0.069(17)
Ag4	0.084(6)	0.084(6)	0.060(5)	0.017(5)	0.017(5)	0.024(7)
Ga1	0.0206(7)	0.0206(7)	0.0206(7)	0.000	0.000	0.000
Se1	0.0276(5)	0.0276(5)	0.0276(5)	0.0050(3)	-0.0050(3)	0.0050(3)
Se2	0.0642(17)	0.0642(17)	0.0642(17)	0.000	0.000	0.000
Se3	0.098(4)	0.098(4)	0.098(4)	0.000	0.000	0.000
			**#7**			
Ag1	0.11(2)	0.11(2)	0.037(2)	-0.017(8)	-0.017(8)	0.06(2)
Ag2	0.41(9)	0.048(11)	0.41(9)	0.000	-0.15(7)	0.000
Ag3	0.21(3)	0.082(13)	0.24(4)	-0.078(16)	-0.03(3)	-0.051(19)
Ag4	0.078(5)	0.078(5)	0.057(6)	0.013(5)	0.013(5)	0.003(7)
Ga1	0.0209(7)	0.0209(7)	0.0209(7)	0.000	0.000	0.000
Se1	0.0290(4)	0.0290(4)	0.0290(4)	0.0050(3)	-0.0050(3)	0.0050(3)
Se2	0.0611(15)	0.0611(15)	0.0611(15)	0.000	0.000	0.000
Se3	0.104(3)	0.104(3)	0.104(3)	0.000	0.000	0.000
			**#8**			
Ag1	0.093(16)	0.093(16)	0.034(2)	-0.012(5)	-0.012(5)	0.042(16)
Ag2	0.221(17)	0.22(2)	0.221(17)	0.000	-0.004(10)	0.000
Ag3	0.19(3)	0.122(17)	0.34(4)	-0.14(2)	0.00(3)	-0.06(2)
Ag4	0.076(6)	0.076(6)	0.061(7)	0.018(5)	0.018(5)	0.015(8)
Ga1	0.0210(7)	0.0210(7)	0.0210(7)	0.000	0.000	0.000
Se1	0.0284(5)	0.0284(5)	0.0284(5)	0.0046(3)	-0.0046(3)	0.0046(3)
Se2	0.0647(18)	0.0647(18)	0.0647(18)	0.000	0.000	0.000
Se3	0.100(4)	0.100(4)	0.100(4)	0.000	0.000	0.000
			**#9**			
Ag1	0.10(2)	0.10(2)	0.039(2)	-0.008(6)	-0.008(6)	0.04(2)
Ag2	0.23(5)	0.030(12)	0.23(5)	0.000	-0.16(4)	0.000
Ag3	0.19(2)	0.120(14)	0.37(3)	-0.146(17)	-0.02(3)	-0.051(17)
Ag4	0.079(5)	0.079(5)	0.057(5)	0.019(4)	0.019(4)	0.021(6)
Ga1	0.0239(7)	0.0239(7)	0.0239(7)	0.000	0.000	0.000
Se1	0.0311(4)	0.0311(4)	0.0311(4)	0.0048(3)	-0.0048(3)	0.0048(3)
Se2	0.0662(15)	0.0662(15)	0.0662(15)	0.000	0.000	0.000
Se3	0.097(3)	0.097(3)	0.097(3)	0.000	0.000	0.000

Supplementary Table 10. Crystallographic data and refinement details of single crystals as-grown inside the ingots obtained under different hot-press pressures #**n** (**n** = 30, 40, 50, 52.5, 55, 57.5, 70 and 90 MPa). The CCDC deposition numbers are 2170787 to 2170802.

	**#30-1**	**#30-2**	**#40-1**
**Empirical formula**	Ag_9GaSe_6	Ag_9GaSe_6	Ag_9GaSe_6
**formula weight**	1514.31	1514.31	1514.31
**crystal system**	Cubic	Cubic	Cubic
**crystal color**	Black	Black	Black
**space group**	$F\bar{4}3m$	$F\bar{4}3m$	$F\bar{4}3m$
**$a = b = c$ (Å)**	11.1327(11)	11.124(4)	11.1204(3)
**$\alpha = \beta = \gamma$ (°)**	90.00	90.00	90.00
**V (Å^3)**	1379.8(2)	1376.3(8)	1375.19(6)
**Z**	4	4	4
**ρ_{cal} (g/cm^3)**	7.290	7.308	7.314
**μ (mm^{-1})**	30.179	30.254	30.280
**GOOF on F^2**	1.085	1.061	1.111
**R_1, wR_2 (I >2$\sigma(I)$) [a]**	0.0698, 0.1697	0.0712, 0.1848	0.0534, 0.1412
**R_1, wR_2 (all data)**	0.1063, 0.1976	0.0974, 0.2135	0.0932, 0.1804
**largest diff. peak and hole (e/Å^3)**	2.75, −1.51	1.98, −1.32	2.60, −1.21

	**#40-2**	**#50-1**	**#50-2**
**Empirical formula**	Ag_9GaSe_6	Ag_9GaSe_6	Ag_9GaSe_6
**formula weight**	1514.31	1514.31	1514.31
**crystal system**	Cubic	Cubic	Cubic
**crystal color**	Black	Black	Black
**space group**	$F\bar{4}3m$	$F\bar{4}3m$	$F\bar{4}3m$
**$a = b = c$ (Å)**	11.1212(4)	11.143(3)	11.192(4)
**$\alpha = \beta = \gamma$ (°)**	90.00	90.00	90.00
**V (Å^3)**	1375.48(9)	1383.7(6)	1401.9(8)
**Z**	4	4	4
**ρ_{cal} (g/cm^3)**	7.313	7.269	7.175
**μ (mm^{-1})**	30.273	30.092	29.703
**GOOF on F^2**	1.087	1.125	1.048
**R_1, wR_2 (I >2$\sigma(I)$) [a]**	0.0524, 0.1286	0.0607, 0.1467	0.0528, 0.1350
**R_1, wR_2 (all data)**	0.0954, 0.1562	0.0823, 0.1659	0.0885, 0.1578
**largest diff. peak and hole (e/Å^3)**	2.29, −0.96	2.50, −2.39	2.10, −1.53

	**#52.5-1**	**#52.5-2**	**#55-1**
**Empirical formula**	Ag_9GaSe_6	Ag_9GaSe_6	Ag_9GaSe_6
**formula weight**	1514.31	1514.31	1514.31
**crystal system**	Cubic	Cubic	Cubic
**crystal color**	Black	Black	Black
**space group**	$F\bar{4}3m$	$F\bar{4}3m$	$F\bar{4}3m$
**$a = b = c$ (Å)**	11.1320(15)	11.1333(15)	11.149 (2)
**$\alpha = \beta = \gamma$ (°)**	90.00	90.00	90.00
**V (Å^3)**	1379.5(3)	1380.0(3)	1385.9(5)
**Z**	4	4	4
**ρ_{cal} (g/cm^3)**	7.291	7.289	7.257
**μ (mm^{-1})**	30.185	30.174	30.045
**GOOF on F^2**	1.042	1.048	1.084
**R_1, wR_2 (I >2$\sigma(I)$) [a]**	0.0579, 0.1526	0.0495, 0.1329	0.0659, 0.1757
**R_1, wR_2 (all data)**	0.0840, 0.1739	0.0827, 0.1537	0.0919, 0.1980
**largest diff. peak and hole (e/Å^3)**	2.53, −1.27	2.64, −1.28	2.26/-1.27

	**#55-2**	**#57.5-1**	**#57.5-2**

**Empirical formula**	Ag_9GaSe_6	Ag_9GaSe_6	Ag_9GaSe_6
**formula weight**	1514.31	1514.31	1514.31
**crystal system**	Cubic	Cubic	Cubic
**crystal color**	Black	Black	Black
**space group**	$F\bar{4}3m$	$F\bar{4}3m$	$F\bar{4}3m$
$a = b = c$ **(Å)**	11.1149(17)	11.118(2)	11.1216(6)
$\alpha = \beta = \gamma$ **(°)**	90.00	90.00	90.00
V **(Å^3)**	1373.1(4)	1374.3(5)	1375.63(13)
Z	4	4	4
ρ_{cal} **(g/cm^3)**	7.325	7.319	7.312
μ **(mm^{-1})**	30.325	30.299	30.270
**GOOF on** F^2	1.082	1.103	1.073
R_1, wR_2 ($I > 2\sigma(I)$) [a]	0.0507, 0.1572	0.0648, 0.1757	0.0683, 0.1829
R_1, wR_2 **(all data)**	0.0885, 0.1777	0.0861, 0.1909	0.0816, 0.1951
**largest diff. peak and hole (e/Å^3)**	2.46, −1.28	2.72/-1.42	3.02/-1.47

	**#75-1**	**#75-2**	**#90-1**
**Empirical formula**	Ag_9GaSe_6	Ag_9GaSe_6	Ag_9GaSe_6
**formula weight**	1514.31	1514.31	1514.31
**crystal system**	Cubic	Cubic	Cubic
**crystal color**	Black	Black	Black
**space group**	$F\bar{4}3m$	$F\bar{4}3m$	$F\bar{4}3m$
$a = b = c$ **(Å)**	11.1353(14)	11.1330(19)	11.133(2)
$\alpha = \beta = \gamma$ **(°)**	90.00	90.00	90.00
V **(Å^3)**	1380.7(3)	1379.9(4)	1379.8(5)
Z	4	4	4
ρ_{cal} **(g/cm^3)**	7.285	7.289	7.290
μ **(mm^{-1})**	30.158	30.177	30.178
**GOOF on** F^2	1.054	1.110	1.146
R_1, wR_2 ($I > 2\sigma(I)$) [a]	0.0541, 0.1337	0.0652, 0.1757	0.0519, 0.1200
R_1, wR_2 **(all data)**	0.0951, 0.1562	0.0928, 0.2021	0.0813, 0.1372
**largest diff. peak and hole (e/Å^3)**	2.10, −1.27	2.27, −2.12	2.51, −2.20

	**#90-2**
**Empirical formula**	Ag_9GaSe_6
**formula weight**	1514.31
**crystal system**	Cubic
**crystal color**	Black
**space group**	$F\bar{4}3m$
$a = b = c$ **(Å)**	11.178(5)
$\alpha = \beta = \gamma$ **(°)**	90.00
V **(Å^3)**	1396.7(10)
Z	4
ρ_{cal} **(g/cm^3)**	7.201
μ **(mm^{-1})**	29.812
**GOOF on** F^2	1.098
R_1, wR_2 ($I > 2\sigma(I)$) [a]	0.0534, 0.1374
R_1, wR_2 **(all data)**	0.0773, 0.1550
**largest diff. peak and hole (e/Å^3)**	2.51, −1.68

[a] $R_1 = \Sigma||F_o| - |F_c||/\Sigma|F_o|$, $wR_2 = [\Sigma w(F_o^2 - F_c^2)^2/\Sigma w(F_o^2)^2]^{1/2}$

Supplementary Table 11. Atomic coordinates and equivalent isotropic displacement parameters and occupancies of single crystals as-grown inside the ingots obtained under different hot-press pressures #**n** (**n** = 30, 40, 50, 52.5, 55, 57.5, 70 and 90 MPa).

Atom site	*x*	*y*	*z*	*Occu.*	*U*eq/Å^2	Wyck.
			**#30-1**			
Ag1	0.25000	0.25000	0.03330	0.455	0.095	24*g*
Ag2	0.29600	0.00000	0.50000	0.170	0.410	24*f*
Ag3	0.34800	0.15200	0.53400	0.230	0.175	48*h*
Ag4	0.31810	0.18190	0.47820	0.200	0.075	48*h*
Ga1	0.00000	0.00000	0.50000	1.000	0.021	4*b*
Se1	0.12350	0.12350	0.62350	1.000	0.028	16*e*
Se2	0.25000	0.25000	0.25000	1.000	0.065	4*c*
Se3	0.00000	0.50000	0.50000	1.000	0.097	4*a*
			**#30-2**			
Ag1	0.25000	0.25000	0.03300	0.466	0.106	24*g*
Ag2	0.29600	0.00000	0.50000	0.103	0.118	24*f*
Ag3	0.35250	0.14750	0.55200	0.201	0.126	48*h*
Ag4	0.32090	0.17910	0.48070	0.264	0.083	48*h*
Ga1	0.00000	0.00000	0.50000	1.000	0.023	4*b*
Se1	0.12370	0.12370	0.62370	1.000	0.030	16*e*
Se2	0.25000	0.25000	0.25000	1.000	0.066	4*c*
Se3	0.00000	0.50000	0.50000	1.000	0.098	4*a*
			**#40-1**			
Ag1	0.25000	0.25000	0.03300	0.457	0.099	24*g*
Ag2	0.29700	0.00000	0.50000	0.170	0.360	24*f*
Ag3	0.34710	0.15290	0.53400	0.250	0.195	48*h*
Ag4	0.31750	0.18250	0.47920	0.186	0.076	48*h*
Ga1	0.00000	0.00000	0.50000	1.000	0.025	4*b*
Se1	0.12364	0.12364	0.62364	1.000	0.032	16*e*
Se2	0.25000	0.25000	0.25000	1.000	0.070	4*c*
Se3	0.00000	0.50000	0.50000	1.000	0.100	4*a*
			**#40-2**			
Ag1	0.25000	0.25000	0.03270	0.484	0.106	24*g*
Ag2	0.29600	0.00000	0.50000	0.154	0.320	24*f*
Ag3	0.35020	0.14980	0.54400	0.224	0.172	48*h*
Ag4	0.32030	0.17970	0.47870	0.207	0.076	48*h*
Ga1	0.00000	0.00000	0.50000	1.000	0.025	4*b*
Se1	0.12338	0.12338	0.62338	1.000	0.032	16*e*
Se2	0.25000	0.25000	0.25000	1.000	0.070	4*c*
Se3	0.00000	0.50000	0.50000	1.000	0.098	4*a*
			**#50-1**			
Ag1	0.25000	0.25000	0.03350	0.452	0.092	24*g*
Ag2	0.29800	0.00000	0.50000	0.125	0.230	24*f*

Ag3	0.34670	0.15330	0.53200	0.274	0.210	48*h*
Ag4	0.31650	0.18350	0.47960	0.188	0.071	48*h*
Ga1	0.00000	0.00000	0.50000	1.000	0.020	4*b*
Se1	0.12335	0.12335	0.62335	1.000	0.027	16*e*
Se2	0.25000	0.25000	0.25000	1.000	0.064	4*c*
Se3	0.00000	0.50000	0.50000	1.000	0.095	4*a*
			**#50-2**			
Ag1	0.25000	0.25000	0.03350	0.448	0.095	24*g*
Ag2	0.29900	0.00000	0.50000	0.180	0.380	24*f*
Ag3	0.34710	0.15290	0.53600	0.240	0.189	48*h*
Ag4	0.31790	0.18210	0.47870	0.200	0.078	48*h*
Ga1	0.00000	0.00000	0.50000	1.000	0.024	4*b*
Se1	0.12344	0.12344	0.62344	1.000	0.031	16*e*
Se2	0.25000	0.25000	0.25000	1.000	0.069	4*c*
Se3	0.00000	0.50000	0.50000	1.000	0.100	4*a*
			**#52.5-1**			
Ag1	0.25000	0.25000	0.03340	0.448	0.093	24*g*
Ag2	0.29400	0.00000	0.50000	0.097	0.190	24*f*
Ag3	0.34680	0.15320	0.53000	0.284	0.199	48*h*
Ag4	0.31680	0.18320	0.47820	0.193	0.074	48*h*
Ga1	0.00000	0.00000	0.50000	1.000	0.024	4*b*
Se1	0.12359	0.12359	0.62359	1.000	0.030	16*e*
Se2	0.25000	0.25000	0.25000	1.000	0.068	4*c*
Se3	0.00000	0.50000	0.50000	1.000	0.097	4*a*
			**#52.5-2**			
Ag1	0.25000	0.25000	0.03330	0.448	0.093	24*g*
Ag2	0.29800	0.00000	0.50000	0.149	0.290	24*f*
Ag3	0.34840	0.15160	0.53700	0.246	0.184	48*h*
Ag4	0.31720	0.18280	0.47880	0.206	0.074	48*h*
Ga1	0.00000	0.00000	0.50000	1.000	0.021	4*b*
Se1	0.12359	0.12359	0.62359	1.000	0.028	16*e*
Se2	0.25000	0.25000	0.25000	1.000	0.066	4*c*
Se3	0.00000	0.50000	0.50000	1.000	0.098	4*a*
			**#55-1**			
Ag1	0.25000	0.25000	0.03380	0.425	0.088	24*g*
Ag2	0.29300	0.00000	0.50000	0.160	0.360	24*f*
Ag3	0.34700	0.15300	0.53000	0.250	0.193	48*h*
Ag4	0.31630	0.18370	0.47840	0.200	0.077	48*h*
Ga1	0.00000	0.00000	0.50000	1.000	0.023	4*b*
Se1	0.12353	0.12353	0.62353	1.000	0.029	16*e*
Se2	0.25000	0.25000	0.25000	1.000	0.065	4*c*
Se3	0.00000	0.50000	0.50000	1.000	0.101	4*a*
			**#55-2**			
Ag1	0.25000	0.25000	0.03320	0.426	0.089	24*g*

Ag2	0.29600	0.00000	0.50000	0.137	0.250	24*f*
Ag3	0.34540	0.15460	0.53100	0.267	0.198	48*h*
Ag4	0.31580	0.18420	0.47870	0.201	0.078	48*h*
Ga1	0.00000	0.00000	0.50000	1.000	0.023	4*b*
Se1	0.12332	0.12332	0.62332	1.000	0.030	16*e*
Se2	0.25000	0.25000	0.25000	1.000	0.067	4*c*
Se3	0.00000	0.50000	0.50000	1.000	0.098	4*a*
			**#57.5-1**			
Ag1	0.25000	0.25000	0.03360	0.437	0.091	24*g*
Ag2	0.29600	0.00000	0.50000	0.150	0.340	24*f*
Ag3	0.34580	0.15420	0.52900	0.260	0.185	48*h*
Ag4	0.31580	0.18420	0.47900	0.200	0.076	48*h*
Ga1	0.00000	0.00000	0.50000	1.000	0.021	4*b*
Se1	0.12343	0.12343	0.62343	1.000	0.027	16*e*
Se2	0.25000	0.25000	0.25000	1.000	0.065	4*c*
Se3	0.00000	0.50000	0.50000	1.000	0.097	4*a*
			**#57.5-2**			
Ag1	0.25000	0.25000	0.03400	0.419	0.085	24*g*
Ag2	0.29900	0.00000	0.50000	0.170	0.420	24*f*
Ag3	0.34600	0.15400	0.52900	0.240	0.188	48*h*
Ag4	0.31580	0.18420	0.47860	0.210	0.078	48*h*
Ga1	0.00000	0.00000	0.50000	1.000	0.021	4*b*
Se1	0.12336	0.12336	0.62336	1.000	0.028	16*e*
Se2	0.25000	0.25000	0.25000	1.000	0.064	4*c*
Se3	0.00000	0.50000	0.50000	1.000	0.099	4*a*
			**#75-1**			
Ag1	0.25000	0.25000	-0.03360	0.429	0.097	24*g*
Ag2	0.00000	0.00000	-0.17890	0.176	0.290	24*f*
Ag3	0.17200	0.07300	0.07300	0.051	0.140	48*h*
Ag4	0.18210	0.18210	-0.02010	0.192	0.083	48*h*
Ag5	0.15510	0.15510	0.03000	0.204	0.169	48*h*
Ga1	0.50000	0.00000	0.00000	1.000	0.024	4*b*
Se1	0.37684	-0.12316	-0.12316	1.000	0.031	16*e*
Se2	0.25000	0.25000	-0.25000	1.000	0.068	4*d*
Se3	0.00000	0.00000	0.00000	1.000	0.101	4*a*
			**#75-2**			
Ag1	0.25000	0.25000	-0.03420	0.389	0.085	24*g*
Ag2	0.00000	0.00000	-0.17900	0.190	0.280	24*f*
Ag3	0.16100	0.09700	0.09700	0.110	0.170	48*h*
Ag4	0.18580	0.18580	-0.02140	0.200	0.087	48*h*
Ag5	0.15800	0.15800	0.01300	0.150	0.130	48*h*
Ga1	0.50000	0.00000	0.00000	1.000	0.023	4*b*
Se1	0.37657	-0.12343	-0.12343	1.000	0.030	16*e*
Se2	0.25000	0.25000	-0.25000	1.000	0.066	4*d*

Se3	0.00000	0.00000	0.00000	1.000	0.102	4*a*
			**#90-1**			
Ag1	0.25000	0.25000	-0.03360	0.408	0.085	24*g*
Ag2	0.00000	0.00000	-0.17948	0.173	0.280	24*f*
Ag3	0.17300	0.07400	0.07400	0.034	0.088	48*h*
Ag4	0.17960	0.17960	-0.02150	0.267	0.081	48*h*
Ag5	0.14980	0.14980	0.04800	0.159	0.106	48*h*
Ga1	0.50000	0.00000	0.00000	1.000	0.021	4*b*
Se1	0.37663	-0.12337	-0.12337	1.000	0.028	16*e*
Se2	0.25000	0.25000	-0.25000	1.000	0.064	4*d*
Se3	0.00000	0.00000	0.00000	1.000	0.097	4*a*
			**#90-2**			
Ag1	0.25000	0.25000	-0.03360	0.402	0.083	24*g*
Ag2	0.00000	0.00000	-0.17970	0.220	0.390	24*f*
Ag3	0.15900	0.09400	0.09400	0.046	0.092	48*h*
Ag4	0.18060	0.18060	-0.02240	0.257	0.080	48*h*
Ag5	0.15190	0.15190	0.03900	0.136	0.094	48*h*
Ga1	0.50000	0.00000	0.00000	1.000	0.022	4*b*
Se1	0.37648	-0.12352	-0.12352	1.000	0.029	16*e*
Se2	0.25000	0.25000	-0.25000	1.000	0.066	4*d*
Se3	0.00000	0.00000	0.00000	1.000	0.099	4*a*

Supplementary Table 12. Anisotropic Displacement Parameters (Å^2) of single crystals as-grown inside the ingots obtained under different hot-press pressures #**n** (**n** = 30, 40, 50, 52.5, 55, 57.5, 70 and 90 MPa). The anisotropic displacement factor exponent takes the form: $-2\pi^2$ ($U_{11}h^2a^{*2}$ + $U_{22}k^2b^{*2}$ + $U_{33}l^2c^{*2}$ + $2U_{23}klb^*c^*$+ $2U_{13}hla^*c^*$ + $2U_{12}hka^*b^*$).

Atom site	U_{11}	U_{22}	U_{33}	U_{23}	U_{13}	U_{12}
			**#30-1**			
Ag1	0.126(8)	0.126(8)	0.033(2)	0.000	0.000	0.066(9)
Ag2	0.065(19)	0.580(150)	0.580(150)	-0.280(120)	0.000	0.000
Ag3	0.124(13)	0.124(13)	0.280(50)	-0.070(15)	0.070(15)	0.035(13)
Ag4	0.086(6)	0.086(6)	0.053(6)	0.024(5)	-0.024(5)	-0.025(7)
Ga1	0.021(8)	0.021(8)	0.021(8)	0.000	0.000	0.000
Se1	0.0278(5)	0.0278(5)	0.0278(5)	-0.0049(3)	-0.0049(3)	-0.0049(3)
Se2	0.065(2)	0.065(2)	0.065(2)	0.000	0.000	0.000
Se3	0.097(4)	0.097(4)	0.097(4)	0.000	0.000	0.000
			**#30-2**			
Ag1	0.140(10)	0.140(10)	0.037(2)	0.000	0.000	0.089(11)
Ag2	0.072(15)	0.140(14)	0.140(14)	-0.010(19)	0.000	0.000
Ag3	0.116(9)	0.116(9)	0.148(17)	-0.020(7)	0.020(7)	0.046(9)
Ag4	0.089(5)	0.089(5)	0.071(6)	0.010(4)	-0.010(4)	-0.011(6)
Ga1	0.0231(9)	0.0231(9)	0.0231(9)	0.000	0.000	0.000
Se1	0.0298(6)	0.0298(6)	0.0298(6)	-0.0045(4)	-0.0045(4)	-0.0045(4)
Se2	0.066(2)	0.066(2)	0.066(2)	0.000	0.000	0.000
Se3	0.098(4)	0.098(4)	0.098(4)	0.000	0.000	0.000
			**#40-1**			
Ag1	0.130(8)	0.130(8)	0.039(2)	0.000	0.000	0.073(8)
Ag2	0.069(14)	0.510(130)	0.510(130)	-0.220(90)	0.000	0.000
Ag3	0.130(10)	0.130(10)	0.330(40)	-0.076(12)	0.076(12)	0.031(11)
Ag4	0.087(6)	0.087(6)	0.053(5)	0.019(4)	-0.019(4)	-0.028(6)
Ga1	0.0250(7)	0.0250(7)	0.0250(7)	0.000	0.000	0.000
Se1	0.0323(4)	0.0323(4)	0.0323(4)	-0.0046(3)	-0.0046(3)	-0.0046(3)
Se2	0.0705(17)	0.0705(17)	0.0705(17)	0.000	0.000	0.000
Se3	0.100(3)	0.100(3)	0.100(3)	0.000	0.000	0.000
			**#40-2**			
Ag1	0.139(8)	0.139(8)	0.0392(18)	0.000	0.000	0.083(8)
Ag2	0.049(11)	0.460(90)	0.460(90)	-0.200(70)	0.000	0.000
Ag3	0.128(9)	0.128(9)	0.260(40)	-0.054(12)	-0.054(12)	0.043(10)
Ag4	0.084(4)	0.084(4)	0.058(4)	0.019(3)	-0.019(3)	-0.015(5)
Ga1	0.0254(7)	0.0254(7)	0.0254(7)	0.000	0.000	0.000
Se1	0.0317(4)	0.0317(4)	0.0317(4)	-0.0049(3)	-0.0049(3)	-0.0049(3)
Se2	0.0699(15)	0.0699(15)	0.0699(15)	0.000	0.000	0.000
Se3	0.098(3)	0.098(3)	0.098(3)	0.000	0.000	0.000
			**#50-1**			

Ag1	0.122(7)	0.122(7)	0.0318(18)	0.000	0.000	0.069(8)
Ag2	0.045(13)	0.320(60)	0.320(60)	-0.080(50)	0.000	0.000
Ag3	0.134(8)	0.134(8)	0.330(30)	-0.083(10)	0.083(10)	0.032(10)
Ag4	0.084(5)	0.084(5)	0.046(4)	0.021(3)	-0.021(3)	-0.028(5)
Ga1	0.0197(6)	0.0197(6)	0.0197(6)	0.000	0.000	0.000
Se1	0.0268(4)	0.0268(4)	0.0268(4)	-0.0047(2)	-0.0047(2)	-0.0047(2)
Se2	0.0641(14)	0.0641(14)	0.0641(14)	0.000	0.000	0.000
Se3	0.095(3)	0.095(3)	0.095(3)	0.000	0.000	0.000
			**#50-2**			
Ag1	0.123(7)	0.123(7)	0.0382(19)	0.000	0.000	0.065(7)
Ag2	0.065(12)	0.530(120)	0.530(120)	-0.160(80)	0.000	0.000
Ag3	0.124(10)	0.124(10)	0.320(40)	-0.077(12)	0.077(12)	0.026(10)
Ag4	0.091(5)	0.091(5)	0.053(4)	0.021(4)	-0.021(4)	-0.030(6)
Ga1	0.0244(7)	0.0244(7)	0.0244(7)	0.000	0.000	0.000
Se1	0.0311(4)	0.0311(4)	0.0311(4)	-0.0050(3)	-0.0050(3)	-0.0050(3)
Se2	0.0691(15)	0.0691(15)	0.0691(15)	0.000	0.000	0.000
Se3	0.100(3)	0.100(3)	0.100(3)	0.000	0.000	0.000
			**#52.5-1**			
Ag1	0.121(7)	0.121(7)	0.036(2)	0.000	0.000	0.061(8)
Ag2	0.035(11)	0.270(60)	0.270(60)	-0.140(60)	0.000	0.000
Ag3	0.145(9)	0.145(9)	0.310(30)	-0.079(11)	0.079(11)	0.037(10)
Ag4	0.088(6)	0.088(6)	0.046(4)	0.019(3)	-0.019(3)	-0.024(6)
Ga1	0.0235(7)	0.0235(7)	0.0235(7)	0.000	0.000	0.000
Se1	0.0297(4)	0.0297(4)	0.0297(4)	-0.0050(3)	-0.0050(3)	-0.0050(3)
Se2	0.0676(16)	0.0676(16)	0.0676(16)	0.000	0.000	0.000
Se3	0.097(3)	0.097(3)	0.097(3)	0.000	0.000	0.000
			**#52.5-2**			
Ag1	0.121(7)	0.121(7)	0.0357(17)	0.000	0.000	0.097(9)
Ag2	0.053(11)	0.420(90)	0.420(90)	-0.160(70)	0.000	0.000
Ag3	0.128(9)	0.128(9)	0.300(30)	-0.071(11)	0.071(11)	0.038(10)
Ag4	0.086(5)	0.086(5)	0.048(4)	0.016(3)	-0.016(3)	-0.024(5)
Ga1	0.0208(6)	0.0208(6)	0.0208(6)	0.000	0.000	0.000
Se1	0.0282(4)	0.0282(4)	0.0282(4)	-0.0047(2)	-0.0047(2)	-0.0047(2)
Se2	0.0663(14)	0.0663(14)	0.0663(14)	0.000	0.000	0.000
Se3	0.098(3)	0.098(3)	0.098(3)	0.000	0.000	0.000
			**#55-1**			
Ag1	0.115(7)	0.115(7)	0.034(2)	0.000	0.000	0.055(8)
Ag2	0.059(16)	0.510(140)	0.510(140)	-0.260(110)	0.000	0.000
Ag3	0.139(12)	0.139(12)	0.300(40)	-0.084(14)	0.084(14)	0.029(13)
Ag4	0.091(6)	0.091(6)	0.049(5)	0.020(4)	-0.020(4)	-0.029(7)
Ga1	0.0233(7)	0.0233(7)	0.0233(7)	0.000	0.000	0.000
Se1	0.0298(4)	0.0298(4)	0.0298(4)	-0.0048(3)	-0.0048(3)	-0.0048(3)
Se2	0.0654(17)	0.0654(17)	0.0654(17)	0.000	0.000	0.000
Se3	0.101(4)	0.101(4)	0.101(4)	0.000	0.000	0.000

			#55-2			
Ag1	0.117(7)	0.117(7)	0.0325(19)	0.000	0.000	0.061(7)
Ag2	0.050(11)	0.350(80)	0.350(80)	-0.130(60)	0.000	0.000
Ag3	0.129(8)	0.129(8)	0.340(30)	-0.076(10)	0.076(10)	0.034(9)
Ag4	0.092(6)	0.092(6)	0.050(4)	0.019(3)	-0.019(3)	-0.032(7)
Ga1	0.0225(7)	0.0225(7)	0.0225(7)	0.000	0.000	0.000
Se1	0.0295(4)	0.0295(4)	0.0295(4)	-0.0044(3)	-0.0044(3)	-0.0044(3)
Se2	0.0667(15)	0.0667(15)	0.0667(15)	0.000	0.000	0.000
Se3	0.098(3)	0.098(3)	0.098(3)	0.000	0.000	0.000
			#57.5-1			
Ag1	0.120(8)	0.120(8)	0.0324(19)	0.000	0.000	0.066(8)
Ag2	0.063(15)	0.480(140)	0.480(140)	-0.210(110)	0.000	0.000
Ag3	0.121(9)	0.121(9)	0.310(30)	-0.080(12)	0.080(12)	0.021(10)
Ag4	0.088(6)	0.088(6)	0.052(5)	0.022(4)	-0.022(4)	-0.032(7)
Ga1	0.0207(6)	0.0207(6)	0.0207(6)	0.000	0.000	0.000
Se1	0.0274(4)	0.0274(4)	0.0274(4)	-0.0049(3)	-0.0049(3)	-0.0049(3)
Se2	0.0653(16)	0.0653(16)	0.0653(16)	0.000	0.000	0.000
Se3	0.097(3)	0.097(3)	0.097(3)	0.000	0.000	0.000
			#57.5-2			
Ag1	0.111(7)	0.111(7)	0.0315(19)	0.000	0.000	0.053(7)
Ag2	0.080(20)	0.590(190)	0.590(190)	-0.230(130)	0.000	0.000
Ag3	0.127(13)	0.127(13)	0.310(40)	-0.082(14)	-0.082(14)	0.024(13)
Ag4	0.093(6)	0.093(6)	0.049(5)	0.017(4)	-0.017(4)	-0.031(7)
Ga1	0.0214(6)	0.0214(6)	0.0214(6)	0.000	0.000	0.000
Se1	0.0277(4)	0.0277(4)	0.0277(4)	-0.0044(3)	-0.0044(3)	-0.0044(3)
Se2	0.0643(16)	0.0643(16)	0.0643(16)	0.000	0.000	0.000
Se3	0.099(4)	0.099(4)	0.099(4)	0.000	0.000	0.000
			#75-1			
Ag1	0.128(4)	0.128(4)	0.0352(18)	0.000	0.000	0.075(5)
Ag2	0.410(60)	0.410(60)	0.042(9)	0.000	0.000	-0.200(70)
Ag3	0.040(20)	0.190(60)	0.190(60)	-0.020(60)	-0.030(20)	-0.030(20)
Ag4	0.094(5)	0.094(5)	0.060(5)	0.030(4)	0.030(4)	0.033(6)
Ag5	0.109(9)	0.109(9)	0.290(40)	-0.062(12)	-0.062(12)	-0.027(12)
Ga1	0.0241(7)	0.0241(7)	0.0241(7)	0.000	0.000	0.000
Se1	0.0305(4)	0.0305(4)	0.0305(4)	-0.0050(3)	-0.0050(3)	-0.0050(3)
Se2	0.0681(15)	0.0681(15)	0.0681(15)	0.000	0.000	0.000
Se3	0.101(3)	0.101(3)	0.101(3)	0.000	0.000	0.000
			#75-2			
Ag1	0.112(8)	0.112(8)	0.031(2)	0.000	0.000	0.060(8)
Ag2	0.400(80)	0.400(80)	0.056(11)	0.000	0.000	-0.160(80)
Ag3	0.060(15)	0.230(60)	0.230(60)	-0.110(60)	-0.013(13)	-0.013(13)
Ag4	0.150(9)	0.105(9)	0.052(5)	0.028(7)	0.028(7)	0.048(10)
Ag5	0.100(14)	0.100(14)	0.190(60)	-0.070(20)	-0.070(20)	-0.009(17)
Ga1	0.0226(7)	0.0226(7)	0.0226(7)	0.000	0.000	0.000

Se1	0.0294(4)	0.0294(4)	0.0294(4)	-0.0046(3)	-0.0046(3)	-0.0046(3)
Se2	0.0663(17)	0.0663(17)	0.0663(17)	0.000	0.000	0.000
Se3	0.102(4)	0.102(4)	0.102(4)	0.000	0.000	0.000
			**#90-1**			
Ag1	0.111(5)	0.111(5)	0.0324(18)	0.000	0.000	0.058(6)
Ag2	0.410(60)	0.410(60)	0.042(9)	0.000	0.000	-0.120(50)
Ag3	0.085(12)	0.090(9)	0.090(9)	-0.001(9)	0.000(6)	0.000(6)
Ag4	0.093(4)	0.093(4)	0.059(4)	0.012(2)	0.012(2)	0.020(4)
Ag5	0.095(8)	0.095(8)	0.128(13)	-0.019(5)	-0.019(5)	-0.032(8)
Ga1	0.0214(6)	0.0214(6)	0.0214(6)	0.000	0.000	0.000
Se1	0.0281(3)	0.0281(3)	0.0281(3)	-0.0049(3)	-0.0049(3)	-0.0049(3)
Se2	0.0644(14)	0.0644(14)	0.0644(14)	0.000	0.000	0.000
Se3	0.097(3)	0.097(3)	0.097(3)	0.000	0.000	0.000
			**#90-2**			
Ag1	0.108(5)	0.108(5)	0.0332(18)	0.000	0.000	0.051(6)
Ag2	0.550(100)	0.550(100)	0.059(10)	0.000	0.000	-0.080(90)
Ag3	0.085(16)	0.096(12)	0.096(12)	0.001(16)	-0.009(10)	-0.009(10)
Ag4	0.094(4)	0.094(4)	0.052(4)	0.014(3)	0.014(3)	0.026(4)
Ag5	0.090(8)	0.090(8)	0.103(14)	-0.017(6)	-0.017(6)	-0.028(8)
Ga1	0.0223(6)	0.0223(6)	0.0223(6)	0.000	0.000	0.000
Se1	0.0289(4)	0.0289(4)	0.0289(4)	-0.0050(3)	-0.0050(3)	-0.0050(3)
Se2	0.0664(15)	0.0664(15)	0.0664(15)	0.000	0.000	0.000
Se3	0.099(3)	0.099(3)	0.099(3)	0.000	0.000	0.000

Supplementary Table 13. Experimental density measured by Archimedes method and the relative density for Ag_9GaSe_6, $Ag_9GaSe_{5.5}Te_{0.5}$ and $Ag_{8.3}Cu_{0.7}GaSe_6$ samples before and after annealing.

Sample	Before/after annealing	Density* (g/cm^3)	Relative density
Ag_9GaSe_6	before	7.18	98.3%
	after	7.16	98.1%
$Ag_9GaSe_{5.5}Te_{0.5}$	before	7.20	98.6%
	after	7.22	98.9%
$Ag_{8.3}Cu_{0.7}GaSe_6$	before	7.07	96.9%
	after	7.08	97.0%

* The density measurement uncertainty is within 1%.

Supplementary Table 14. Typical *p*-type and *n*-type liquid-like thermoelectric materials.

Type	Material	ZT_{max}	Structure type	Type	Material	ZT_{max}	Structure type
	$Cu_{1.8}S$ + 0.75% graphene[6]	1.5 at 873 K			Ag_2S[31]	0.55 at 580 K	
	Cu_2S + 2 mol% In_2S_3[7]	1.2 at 850 K			Ag_2Se[32]	1.2 at 390 K	Ag_2Q
	$Cu_{1.97}S$[8]	1.7 at 1000 K			Ag_2Te + PbTe + Ag[33]	1.0 at 500–600K	
	Cu_2Se[9]	1.6 at 1000 K			Ag_9GaSe_6[1–3,34]	1.6 at 850 K	
	Cu_2Se + 0.75% CNTs[10]	2.4 at 1000 K			$Ag_9GaS_{5.4}Se_{0.6}$[35]	0.6 at 800 K	
	Cu_2Se + 1 mol% In[11]	2.6 at 850 K			$Ag_9Al_{0.96}Cd_{0.04}Se_6$[36]	1.0 at 850 K	argyrodite
	$Cu_2Se_{0.92}S_{0.08}$[12]	2.0 at 1000 K	Cu_2Q		$Ag_8GeSe_{5.88}$[37]	0.55 at 923 K	
	Cu_2Se + 0.6% graphite[13]	2.4 at 850 K			$Ag_8Sn_{1-x}Nb_xSe_6$ ($x \leqslant 0.05$)[38]	1.2 at 850 K	
	Cu_2Te[14]	1.1 at 1000 K					
	$Cu_2S_{0.52}Te_{0.48}$[15]	2.2 at 1000 K					
	$Cu_{1.98}S_{1/3}Se_{1/3}Te_{1/3}$[16]	1.9 at 1000 K					
	$Cu_2Se_{0.7}Te_{0.3}$[17]	1.4 at 1000 K					
p	Cu_7PSe_6[18]	0.35 at 575 K	argyrodite	***n***			
	$Cu_{7.6}Ag_{0.4}GeSe_{5.1}Te_{0.9}$[19]	1.0 at 800 K					
	Cu_5FeS_4 + Cu_2S[20]	1.2 at 900 K					
	$Cu_{4.972}Fe_{0.968}S_4$[21]	0.84 at 675 K	Cu_5FeS_4				
	Cu_5FeS_4[22]	0.62 at 710 K					
	CuAgSe[23]	0.95 at 623 K	CuAgSe				
	CuAgSe[24]	0.9 at 623 K					
	$Ag_{0.96}CrSe_2$[25]	0.5 at 723 K					
	$CuCrSe_2$[26]	1.0 at 773 K	$AgCrSe_2$				
	$(AgCrSe_2)_{0.5}(CuCrSe_2)_{0.5}$[27]	1.4 at 773 K					
	Ag_8GeTe_6[28]	0.48 at 703 K					
	Ag_8SiTe_6[29]	0.48 at 800 K	argyrodite				
	$Ag_9Ga_{0.95}Cd_{0.05}Te_6$[30]	0.65 at 600 K					

References

1. Jiang, B. et al. An argyrodite-type Ag_9GaSe_6 liquid-like material with ultralow thermal conductivity and high thermoelectric performance. *Chem. Commun.* **53**, 11658–11661 (2017).
2. Qi, X. et al. Thermal stability of Ag_9GaSe_6 and its potential as a functionally graded thermoelectric material. *Chem. Eng. J.* **374**, 494–501 (2019).
3. Lin, S. et al. Thermoelectric performance of Ag_9GaSe_6 enabled by low cutoff frequency of acoustic phonons. *Joule* **1**, 816–830 (2017).
4. Zhang, J. et al. Mechanical properties and thermal stability of the high-thermoelectric-performance Cu_2Se compound. *ACS Appl. Mater. Interfaces*, **13**, 45736–45743 (2021).
5. Qiu, P. et al. Suppression of atom motion and metal deposition in mixed ionic electronic conductors. *Nat. Commun.* **9**, 2910 (2018).
6. Tang, H. C. et al. Graphene network in copper sulfide leading to enhanced thermoelectric properties and thermal stability. *Nano Energy* **49**, 267–273 (2018).
7. Meng, Q.-L. et al. Simultaneous enhancement in the power factor and thermoelectric performance of copper sulfide by In_2S_3 doping. *J. Mater. Chem. A* **4**, 12624–12629 (2016).
8. He, Y. et al. High thermoelectric performance in non-toxic earth-abundant copper sulfide. *Adv. Mater.* **26**, 3974–3978 (2014).
9. Liu, H. et al. Copper ion liquid-like thermoelectrics. *Nat. Mater.* **11**, 422–425 (2012).
10. Nunna, R. et al. Ultrahigh thermoelectric performance in Cu_2Se-based hybrid materials with highly dispersed molecular CNTs. *Energy Environ. Sci.* **10**, 1928–1935 (2017).
11. Olvera, A. A. et al. Partial indium solubility induces chemical stability and colossal thermoelectric figure of merit in Cu_2Se. *Energy Environ. Sci.* **10**, 1668–1676 (2017).
12. K. Zhao, et al. Enhanced thermoelectric performance through tuning bonding energy in $Cu_2Se_{1-x}S_x$ liquid-like materials. *Chem. Mater.* **29**, 6367–6377 (2017).
13. Zhao, L. et al. Significant enhancement of figure-of-merit in carbon-reinforced Cu_2Se nanocrystalline solids. *Nano Energy* **41**, 164–171 (2017).
14. He, Y., Zhang, T., Shi, X., Wei, S.-H. & Chen, L. High thermoelectric performance in copper telluride. *NPG Asia Mater.* **7**, e210 (2015).

15. He, Y. et al. Ultrahigh thermoelectric performance in mosaic crystals. *Adv. Mater*. **27**, 3639–3644 (2015).

16. Zhao, K. et al. High thermoelectric performance and low thermal conductivity in $Cu_{2-y}S_{1/3}Se_{1/3}Te_{1/3}$ liquid-like materials with nanoscale mosaic structures. *Nano Energy* **42**, 43–50 (2017).

17. Zhao, K. et al. Thermoelectric properties of $Cu_2Se_{1-x}Te_x$ solid solutions. *J. Mater. Chem. A* **6**, 6977–6986 (2018).

18. Weldert, K. S. et al. Thermoelectric transport in Cu_7PSe_6 with high copper ionic mobility. *J. Am. Chem. Soc.* **136**, 12035–12040 (2014).

19. Jiang, B. et al. Cu_8GeSe_6-based thermoelectric materials with an argyrodite structure. *J. Mater. Chem. C* **5**, 943–952 (2017).

20. Qiu, P. et al. Sulfide bornite thermoelectric material: a natural mineral with ultralow thermal conductivity. *Energy Environ. Sci.* **7**, 4000–4006 (2014).

21. Long, S. O. J. Powell, A. V., Vaqueiro, P. & Hull, S. High thermoelectric performance of bornite through control of the Cu (II) content and vacancy concentration. *Chem. Mater.* **30**, 456–464 (2018).

22. Zhang, A. et al. Twin engineering in solution-synthesized nonstoichiometric Cu_5FeS_4 icosahedral nanoparticles for enhanced thermoelectric performance. *Adv. Funct. Mater.* **28**, 1705117 (2018).

23. Hong, A. J. et al. Anomalous transport and thermoelectric performances of CuAgSe compounds. *Solid State Ion.* **261**, 21–25 (2014).

24. Han, C. et al. Ambient scalable synthesis of surfactant-free thermoelectric CuAgSe nanoparticles with reversible metallic-*n*-*p* conductivity transition. *J. Am. Chem. Soc.* **136**, 17626–17633 (2014).

25. Wu, D. et al. Revisiting $AgCrSe_2$ as a promising thermoelectric material. *Phys. Chem. Chem. Phys.* **18**, 23872–23878 (2016).

26. Bhattacharya, S. et al. $CuCrSe_2$ a high performance phonon glass and electron crystal thermoelectric material. *J. Mater. Chem. A* **1**, 11289–11294 (2013).

27. Bhattacharya, S et al. High thermoelectric performance of $(AgCrSe_2)_{0.5}(CuCrSe_2)_{0.5}$ nano-

composites having all-scale natural hierarchical architectures. *J. Mater. Chem. A* **2**, 17122–17129 (2014).

28. Charoenphakdee, A. et al. Reinvestigation of the thermoelectric properties of Ag_8GeTe_6. *Phys. Stat. Sol. (RRL)* **2**, 65–67 (2008).
29. Charoenphakdee, A. et al. Ag_8SiTe_6: A new thermoelectric material with low thermal conductivity. *JPN. J. Appl. Phys.* **48**, 011603 (2009).
30. Lin, S. et al. Thermoelectric p-type Ag_9GaTe_6 with an intrinsically low lattice thermal conductivity. *ACS Appl. Energy Mater.* **3**, 1892–1898 (2020).
31. Wang, T., Chen, H. -Y., Qiu, P. -F., Shi, X. & Chen, L. -D. Thermoelectric properties of Ag_2S superionic conductor with intrinsically low lattice thermal conductivity. *Acta Phys. Sin.* **68**, 090201 (2019).
32. Yang, D. et al. Facile room temperature solventless synthesis of high thermoelectric performance Ag_2Se via a dissociative adsorption reaction. *J. Mater. Chem. A* **5**, 23243–23251 (2017).
33. Pei, Y., Heinz, N. A. & Snyder, G. J. Alloying to increase the band gap for improving thermoelectric properties of Ag_2Te. *J. Mater. Chem.* **21**, 18256–18260 (2011).
34. Jiang, B. et al. Entropy optimized phase transitions and improved thermoelectric performance in n-type liquid-like Ag_9GaSe_6 materials. *Mater. Today Phys.* **5**, 20–28 (2018).
35. Lin, S. et al. Thermoelectric properties of Ag_9GaS_6 with ultralow lattice thermal conductivity. *Mater. Today Phys.* **6**, 60–67 (2018).
36. Li, W. et al. Crystal structure induced ultralow lattice thermal conductivity in thermoelectric Ag_9AlSe_6. *Adv. Energy Mater.* **8**, 1800030 (2018).
37. Shen, X. et al. High-temperature structural and thermoelectric study of argyrodite Ag_8GeSe_6. *ACS Appl. Mater. Interfaces* **11**, 2168–2176 (2019).
38. Li, W. et al. Low sound velocity contributing to the high thermoelectric performance of Ag_8SnSe_6. *Adv. Sci.* **3**, 1600196 (2016).
